# Cryogenic Vibrational Spectroscopy of the Deprotonated Dimer of Phosphoric Acid

**DOI:** 10.1021/acs.jpca.5c06704

**Published:** 2025-12-15

**Authors:** América Y. Torres-Boy, Jia Han, Gurpur Rakesh D. Prabhu, Martín I. Taccone, Anoushka Ghosh, Hannah Buttkus, Katja Ober, Gerard Meijer, Knut R. Asmis, Anne B. McCoy, Gert von Helden

**Affiliations:** † 28259 Fritz Haber Institute of the Max Planck Society, 14195 Berlin, Germany; ‡ Wilhelm-Ostwald-Institut für Physikalische und Theoretische Chemie, 9180 Universität Leipzig, Linnéstraße 2, 04103 Leipzig, Germany; ¶ Institute of Chemistry and Biochemistry, Freie Universität Berlin, 14195 Berlin, Germany; § Department of Chemistry, 7284 University of Washington, Seattle, Washington 98195, United States

## Abstract

Phosphate-containing molecules are ubiquitous in nature, where they play crucial roles in biochemical processes. Further, they are of technical importance, for example, in certain batteries and in fuel cells, where a unique property of phosphoric acid is exploited—its exceptionally high proton conductivity. Proton transport in phosphoric acid is known to involve proton shuttling; however, the elementary steps involved are not clear. To elucidate the hydrogen bonding preferences of phosphoric acid, we investigate the dihydrogen phosphate anion as well as the deprotonated dimer of phosphoric acid (H_3_PO_4_·H_2_PO_4_^–^) in the gas phase using infrared action spectroscopy in helium nanodroplets and infrared D_2_-tagging photodissociation spectroscopy, and the experimental spectra are compared to theoretical ones. Theory finds for H_3_PO_4_·H_2_PO_4_^–^ two different structures that are predicted to be nearly isoenergetic. The comparison to the experimental spectra, however, allows for a clear assignment and structure identification. The resulting structure has an interesting binding motif, which might be of relevance to interactions of phosphoric acid in the condensed phase and which can serve as a benchmark for quantum chemical calculations.

## Graphical abstract

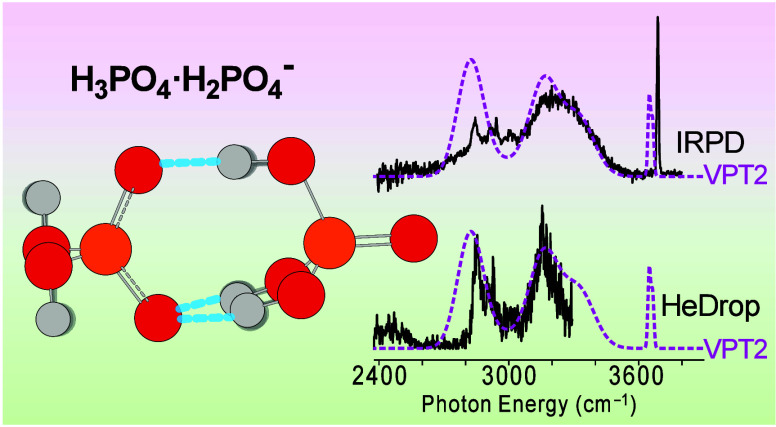

## Introduction

Phosphoric acid and its derivatives occupy a central role in both biological and inorganic chemistry. In biology, phosphate groups are indispensable: they are key constituents of nucleic acids, form the backbone of DNA and RNA, and serve as essential moieties in molecules such as ATP, which is fundamental for cellular energy storage and transfer.^1–3^ Phosphate esters confer extraordinary chemical stability, ensuring the longevity of genetic material, while their negative charge properties facilitate interactions vital for cellular processes such as protein phosphorylation and signal transduction. Cellular regulation, protein function, and even subcellular localization critically depend on the reversible addition or removal of phosphate groups, making phosphorylation a ubiquitous molecular switch in living organisms.

In addition, phosphoric acid (H_3_PO_4_) is important in other fields, ranging from prebiotic chemistry^4^ and atmospheric processes^5^ to the development of fuel cells and batteries.^6–9^ A key property of phosphoric acid is its high proton conductivity,^10–13^ which is the highest of all known liquid substances, and is related to its high degree of autoionization,^10^ which is higher than that of water. For water, its high proton conductivity is partially explained by the famous Grotthuss proton shuttling mechanism.^14^ For phosphoric acid, while it seems likely that a similar mechanism is at hand, the elementary steps are less well understood.^10,15–18^

The study of proton dynamics and hydrogen-bonding motifs in small molecular clusters, particularly neutral, protonated, and deprotonated water clusters, has provided invaluable mechanistic insights.^19,20^ In particular, vibrational spectroscopy, in concert with theoretical calculations, has proven to be a powerful technique for elucidating the structures, binding motifs, and proton-transfer pathways in such clusters and has established benchmark systems for understanding proton localization, shared proton structures, and the role of nuclear quantum effects.^21–23^ A Grotthuss-type proton shuttling mechanism can occur between protonated and neutral as well as between deprotonated and neutral species. *A priori* there is no reason why one of them should be more efficient than the other. For water, however, experiment and theory show that shuttling involving protonated species is faster, and the reason has to do with the unfavorable shape of the proton-accepting orbitals in OH^–^.^14^ This may not be the case for other species, such as phosphoric acid, where deprotonated species could play an important role. An early publication^10^ shows that the remarkable proton conductivity of phosphoric acid is primarily associated with the dihydrogen phosphate anion (H_2_PO_4_^–^). A subsequent study discusses the possible contribution of the deprotonated dimer H_3_PO_4_·H_2_PO_4_^–^ to phosphoric acid conductivity.^24^ Despite this, for phosphoric acid, anionic channels for proton transport seem to be often overlooked.

While there is a vast amount of gas-phase spectroscopic data for organic molecules containing H, N, C, and O atoms, studies for P-containing molecules are rather scarce. This has to do with the circumstance that phosphoric acid is stable in liquid and solid forms, but difficult to vaporize without decomposition.^24–30^ To allow for gas-phase studies on charged species, electrospray ionization can be employed, and phosphate and phosphoric acid containing ionic clusters have been studied using mass spectrometry,^31–35^ vibrational spectroscopy^36–40^ as well as pure theory^12,41–45^ and some of those studies involve theory on pure phosphoric acid clusters.^33,41–45^

In this work, the deprotonated phosphoric acid dimer (dPAD-H_5_) and its fully deuterated counterpart (dPAD-D_5_) are interrogated using two complementary types of cryogenic IR action spectroscopy. As the complexes are cold, spectral congestion is reduced, allowing for a more accurate determination of molecular interactions.

Investigating the hydrogen-bonding motifs in the deprotonated phosphoric acid dimer can help in the understanding of phosphoric acid interactions and contribute to a deeper insight into proton transfer and mobility. Additionally, such spectroscopic studies provide experimental spectra that allow for a direct comparison with predictions using quantum chemistry and can serve as benchmarks for refining theoretical models.

## Experimental Section

IR action spectra of the dihydrogen phosphate anion, as well as of the deprotonated phosphoric acid dimer (H_3_PO_4_·H_2_PO_4_^–^) and its fully deuterated counterpart, are measured using two complementary techniques, in two different experimental setups. The helium droplet setup at the Fritz-Haber-Institut^46–48^ is used to record spectra in liquid helium droplets at a temperature of 0.4 K. At Leipzig University, infrared photodissociation (IRPD) spectra of gas-phase anion complexes tagged with D_2_ are recorded with a cryogenic ion trap triple mass spectrometer.^49,50^ In both experiments, the ions of interest are generated using a nanoelectrospray ion source. Below, some experimental details are given.

### Infrared Spectroscopy in Helium Nanodroplets

Cryogenic IR spectra of dPAD-H_5_ and its fully deuterated counterpart are measured using helium nanodroplets infrared action spectroscopy. The home-built experimental setup has been reported in detail previously.^46–48^ Here, only a brief overview and some specific experimental details are provided. The ions of interest are generated using nanoelectrospray ionization (nESI) from a borosilicate capillary, which is filled with a sample solution of 5 mM phosphoric acid (85%, Sigma-Aldrich Merck, Darmstadt, Germany) in a 1:1 mixture of water and methanol. To substitute exchangeable hydrogen atoms by deuterium atoms, the source region is flooded with D_2_O-saturated nitrogen gas.

After transfer into vacuum, the ions of the desired mass-to-charge ratio (*m*/*z*) are isolated by a quadrupole mass filter. The ions are then deflected 90° by a quadrupole ion bender and transferred into a linear hexapole radio frequency (RF) ion trap. Inside the trap, the ions are confined in the radial direction by the effective RF potential, and in the longitudinal direction by a weak potential (∼5 V) applied to the trap entrance and exit lenses. The housing of the trap is cooled to ∼90 K using cold nitrogen gas. The ions are thermalized within the trap through collisions with precooled helium buffer gas. After filling the trap and after the buffer gas has been pumped out, helium nanodroplets traverse the ion trap. These nanodroplets are generated by the expansion of helium (∼70 bar) through the cryogenic nozzle (19–23 K) of a pulsed Even-Lavie valve operated at a repetition rate of 10 Hz. The size distribution of helium nanodroplets is known to follow a log-normal distribution, and under the experimental conditions used here, this distribution is expected to have a maximum (mode) of ∼5 × 10^4^ and a mean of ∼7 × 10^4^ He atoms.^51^ The droplets travel at a beam velocity of ∼500 m/s^52^ and they can capture an ion through mechanical impact, cooling it to the equilibrium temperature of the droplet (0.4 K). Due to the large mass difference of the ions and the droplets, the ion doped droplets still move at their initial velocity, giving them a kinetic energy of 1.3 meV/amu at 500 m/s. Therefore, even relatively small doped droplets, containing only a few thousand helium atoms, possess sufficient kinetic energy to overcome the longitudinal trapping potential of the ion trap, and as a consequence, ions encapsulated in a helium nanodroplet can exit the trap.

The ion-doped droplets move downstream and interact with the counterpropagating tunable IR light beam of the Fritz Haber Institute infrared free-electron laser (FHI-FEL), which provides IR light in the form of ∼10 μs long macropulses at a 10 Hz repetition rate, consisting of micropulses of ∼5 ps length at a repetition rate of 1 GHz. When an ion inside a helium nanodroplet interacts with the FHI-FEL light at a frequency in resonance with a vibrational transition, the absorption of photons can occur. The photon energy will then be redistributed, first within the molecule and then to the helium environment. This will cause the evaporation of helium atoms and the thermalization of the droplet as well as of the dopant ion back to the equilibrium temperature of 0.4 K. This cooling will occur rapidly compared to the time scale of the FEL macropulse, and a subsequent photon absorption event will occur again from a cold ion in its ground state. Such absorption/thermalization events can occur many times on the time scale of the FHI-FEL macropulse and can lead to the complete evaporation of the droplet. Bare ions can then be detected by a time-of-flight mass spectrometer, and the amount of ions detected depends on the absorption cross-section, the laser fluence, the initial droplet size as well as on the relaxation dynamics.^40^ IR spectra are then obtained by measuring the ion yield as a function of the laser wavelength. Each spectrum is averaged over at least two individual scans.

### Infrared Photodissociation Spectroscopy

Briefly, the deprotonated phosphoric acid dimer is produced in a nanoelectrospray ion source analogous to that used for helium nanodroplets infrared action spectroscopy. The beam of anions is skimmed and collimated in a helium-filled RF-octupole ion guide. Subsequently, the anion complexes are mass-selected using a quadrupole mass filter and guided into an RF ring-electrode ion trap, held at a temperature of 13 K and continuously filled with D_2_ gas. Many collisions of the trapped ions with the buffer gas provide gentle cooling of the internal degrees of freedom close to the ambient temperature. At sufficiently low ion-trap temperatures, ion-messenger complexes are formed via three-body collisions.^53^ Every 100 ms, all ions are extracted from the ion trap and focused, both temporally and spatially, into the center of the extraction region of the orthogonally mounted double-focusing reflectron time-of-flight (TOF) tandem photofragmentation mass spectrometer and detected using the background-free IR^1^MS^2^ detection scheme.^54^ Anions with a particular mass-to-charge ratio (*m*/*z*) are irradiated by a properly timed and widely wavelength tunable IR laser pulse (bandwidth 3.5 cm^–1^). The pulse is supplied by an optical parametric oscillator/amplifier (LaserVision OPO/OPA) laser system pumped by an unseeded Nd:YAG laser (Continuum Surelite EX). IRPD spectra are recorded by monitoring the yields of the irradiated ions and their photofragments, while the laser wavelength is recorded online using a HighFinesse WS6-600 wavelength meter. The wavelength is scanned continuously with a scan speed such that an averaged TOF mass spectrum (over 40 laser shots) is obtained every 2 cm^–1^. Typically, three to five scans are measured and averaged, and the photodissociation cross section is determined as described previously.^49,54^

### Computational Methods

The conformational space is explored using CREST^55^ and the GFN2-xTB method,^56^ resulting in two types of structures, with two structurally similar conformers each. Those, as well as monomeric neutral phosphoric acid (H_3_PO_4_) and dihydrogen phosphate (H_2_PO_4_^–^), are then optimized at a higher level of theory, and their harmonic IR frequencies are calculated at several levels of theory. The Gaussian 16 software package^57^ is used for DFT (B3LYP with GD3BJ^58^ dispersion correction) as well as MP2 (frozen core) calculations. Using the ORCA software package,^59^ double-hybrid with spin-component scaling calculations with the PBEP86 functional and including D4 dispersion correction (revDSD-PBEP86-D4)^60–62^ calculations are performed. All those calculations are performed using the aug-cc-pV­(T+d)­Z basis set.^63^ Including the extra d-function in the aug-cc-pVTZ basis set was observed to have a significant effect on vibrational frequencies. To obtain accurate energetics, Gaussian 16 is used to determine CCSD­(T) single-point energies, also using the aug-cc-pV­(T+d)­Z basis set, at the MP2 minimum erergy structures and for complete Basis Set-Quadratic Becke3 ^64^ (CBS-QB3) calculations.

To go beyond the harmonic approximation, B3LYP IR frequencies are calculated within the GVPT2 anharmonic approximation using Gaussian 16. Geometries are optimized very tightly, the superfine grid is used, and the accuracy of the SCF convergence as well as of the two-electron integrals was increased by a factor of 10.

To plot the calculated IR spectra, the stick spectra are convoluted with Gaussian line shape functions, having a full-width at half-maximum (fwhm) of 0.4% of the wavenumber, which corresponds to the bandwidth of the FHI-FEL laser pulses used in our experiments.

## Results and Discussion

### Experimental IR Spectra of the Deprotonated Phosphoric Acid Dimer

Figure 1 shows the cryogenic IR spectra of the deprotonated phosphoric acid dimer (dPAD-H_5_), of its fully deuterated counterpart (dPAD-D_5_), as well as of the dihydrogen phosphate anion (H_2_PO_4_^–^) in the fingerprint region, recorded using the helium nanodroplet setup. The spectrum of dPAD-H_5_ (top panel) shows a series of mostly narrow and well-resolved bands, labeled from (a) to (j). In the region between 850 and 1000 cm^–1^, four strong bands, labeled (b), (c), (d), and (e), are observed. Bands (b) and (c) are slightly narrower than the other bands in the spectrum. The next strong band (g) at 1095 cm^–1^ is followed by (h), an unexpectedly broad and asymmetric band located at ∼1210 cm^–1^. The highest frequency band observed (j) appears at 1366 cm^–1^. Previous experiments on microhydrated dihydrogen phosphate clusters in the gas phase have assigned bands around 700 and 900 cm^–1^ to the excitation of P—OH stretching vibrations and bands between 1000 and 1350 cm^–1^ to P=O stretching modes.^39^

**Figure 1:**
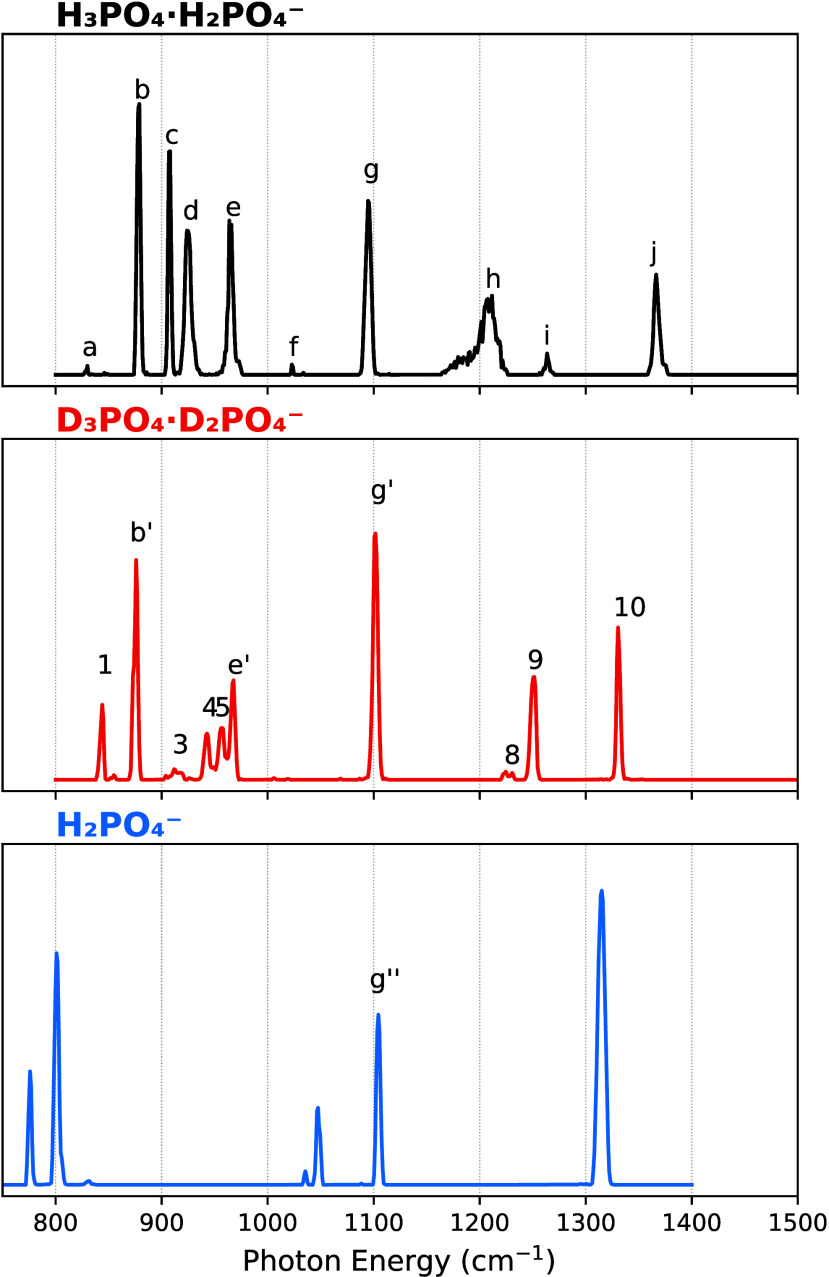
Cryogenic IR action spectra recorded using helium nanodroplets of the deprotonated phosphoric acid dimer, dPAD-H_5_ (top panel), its fully deuterated counterpart, dPAD-D_5_ (middle panel), and the dihydrogen phosphate anion (bottom panel).

The middle panel of Figure 1 shows the IR action spectrum of dPAD-D_5_, which is also composed of a series of narrow bands. They are labeled from (1)-(10) for bands that have no apparent counterpart in the spectrum of dPAD-H_5_, and (b′), (e′), and (g′) for bands that are close in position to bands in the spectrum of dPAD-H_5_.

The spectrum of H_2_PO_4_^–^ (Figure 1, bottom panel) displays very sharp bands as well. The most intense being located at 1315 cm^–1^, two medium intensity bands in the region between 1000 and 1110 cm^–1^, and two rather strong bands between 780 and 810 cm^–1^. An interesting observation is that in all three spectra shown in Figure 1, one band is located at almost the same position, at ∼1105 cm^–1^, which is labeled (g″) in the spectrum of H_2_PO_4_^–^.

The IR spectra of dPAD-H_5_ and dPAD-D_5_ in the O–H (O–D) stretching region are recorded using cryogenic IR spectroscopy in helium nanodroplets as well as using IRPD spectroscopy and are shown in Figure 2. In the O–H stretching region, the helium droplet spectrum spans only up to ∼3300 cm^–1^, due to tuning limitations of the FEL. For dPAD-H_5_, the spectra are composed of broad bands in the range of 2800–3600 cm^–1^ and a sharp band at 3690 cm^–1^. Compared to the IRPD spectrum, the bands observed in the helium droplet spectrum are narrower. For dPAD-D_5_, bands are broad as well, but narrower compared to those in the spectrum of dPAD-H_5_, and located between 2150 and 2600 cm^–1^. Similarly to the dPAD-H_5_ spectrum, a sharp band at the blue side of the spectrum is found at 2720 cm^–1^. Even further to the blue, at 2947 cm^–1^, a weak and slightly broadened band can be observed.

**Figure 2:**
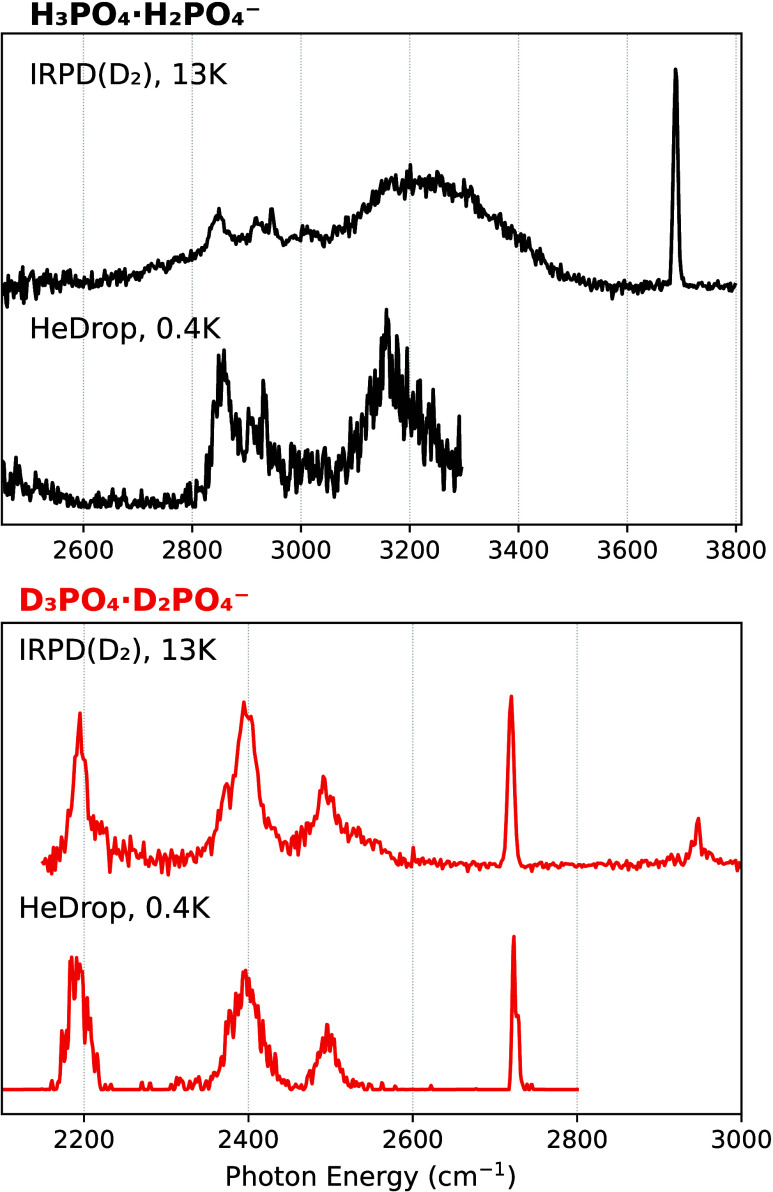
IR photodissociation (IRPD) spectra using D_2_-tagging and spectra recorded using helium nanodroplets (HeDrop) of the deprotonated phosphoric acid dimer, dPAD-H_5_ (top panel), and its fully deuterated counterpart, dPAD-D_5_ (bottom panel).

The IRPD spectrum of D_2_-tagged dPAD-H_5_ in Figure 2 can be compared to the IRPD spectrum of D_2_-tagged H_2_PO_4_^–^ (Figure S1). In that spectrum, two bands are observed, one at 2947 cm^–1^ and one at 3690 cm^–1^. The first band likely corresponds to the excitation of the D–D stretching mode of the tagged molecule (vs 2994 cm^–1^ for free D_2_). This transition gains in IRPD intensity due to the interaction of D_2_ with the H_2_PO_4_^–^ ion. Likewise, the weak band at 2947 cm^–1^ in the IRPD spectrum of dPAD-D_5_ in the lower panel of Figure 2 is assigned to the D_2_ stretching vibration. The band of H_2_PO_4_^–^ at 3690 cm^–1^ is assigned to an in-phase combination of the two free O–H stretching local modes. This band is at nearly the same position as the sharp band in the IRPD spectrum of dPAD-H_5_, so the same assignment is likely.

The two techniques employed differ mainly in three aspects. First, the temperature: using helium nanodroplets, the ions are cooled to 0.4 K, while using D_2_ tagging, the ions are thermalized to a temperature slightly above the ion trap temperature of 13 K. Another important difference is the interaction with the environment, which is almost negligible when using superfluid He, but can have some impact when a tag such as D_2_ interacts with the anion. The third difference is that, while helium nanodroplets require the absorption of multiple photons to yield a signal (sequential one-photon processes), the tagging technique typically requires only one photon and therefore probes the linear absorption regime. The IRPD and helium droplet spectra shown in Figure 2 are similar, suggesting that the influences of tag and helium environment on the spectra are small.

### Quantum Chemistry Calculations

When performing a search through the structural landscape for dPAD-H_5_, two low-energy structure types are found: A and B, represented by structures A1 and B1 in Figure 3. Both structure types are composed of two distinct moieties: phosphoric acid and dihydrogen phosphate, which are connected via hydrogen bonding. In both structure types, the negative charge is delocalized over the two nonprotonated oxygen atoms on the dihydrogen phosphate unit.

**Figure 3:**
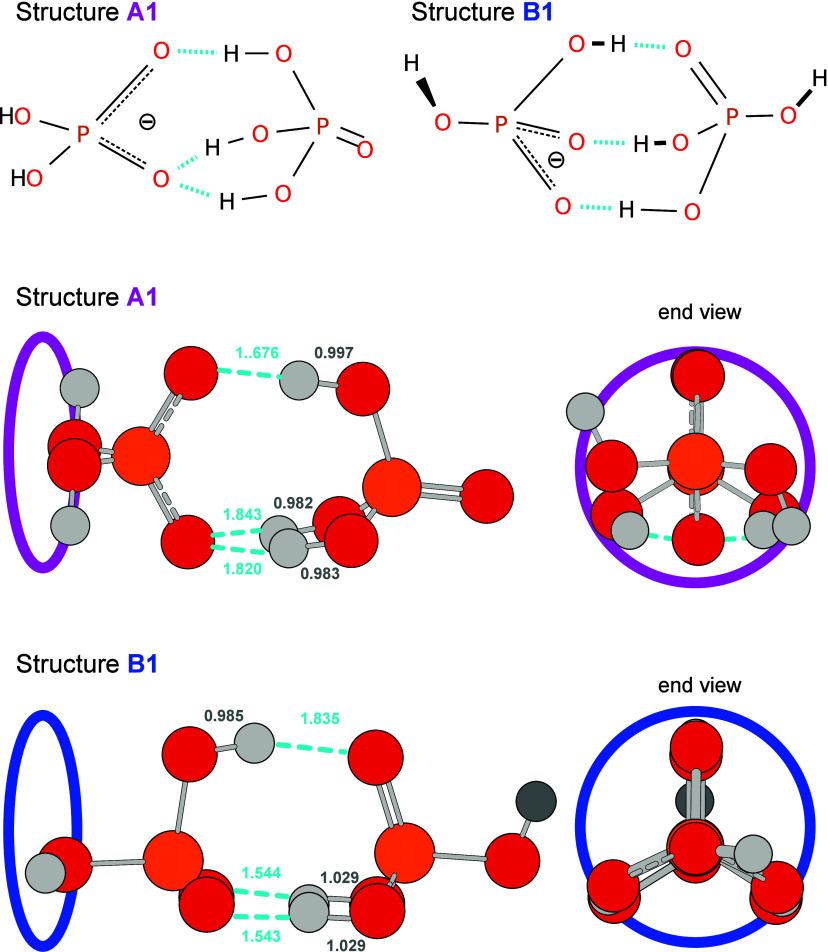
Skeletal-formula depiction and graphic representations of the two lowest energy structures, A1 and B1. The hydrogen-bond lengths (Å) are shown. Their relative energies are sensitive to the level of theory (Table 1). Additional analogous structures that differ by the orientation of the free OH groups are presented in Figure S2, and their relative energy calculated at B3LYP-D3­(BJ)/aug-cc-pV­(T+d)­Z level of theory is summarized in Table S1.

In structure A, the three hydroxyl groups of the phosphoric acid unit point toward the POO^–^ group of dihydrogen phosphate, and the two free −OH groups on dihydrogen phosphate are not involved in H-bonding. Interestingly, two of the three −OH groups involved in H-bonding coordinate to the same O atom of the POO^–^ group. That is, in structure A, the two oxygen atoms of phosphate act as single and double H-bond acceptors, while the phosphoric acid protons are H-bond donors (AA2/DDD). This binding motif and the phosphoric acid geometry of this structure resemble the lowest energy structure calculated for the phosphoric acid-formate anion cluster, studied previously.^40^

The hydrogen-bonding motif in structure B involves three −OH groups as well, with each −OH group having a distinct O atom to coordinate to (DAA/ADD). The two remaining −OH groups are on different molecular units and do not participate in hydrogen bonding.

Structure types A and B each have two structurally very similar conformers, distinguished by the orientation of the free −OH groups (see Figure S2). In structure type A, those conformers differ by trans (A1) or cis (A2) orientation of the free OH groups, and in type B, the −OH group on the formally neutral unit on the right side is rotated by ∼180°. As a side note, in structure B1, the −OH group on the left side can also be rotated by ∼180°, then yielding the chiral mirror image of the structure shown in the figure. The heavy atom positions and bond lengths in those are similar, and so are their calculated IR spectra (Figures S3 and S4). In the following, only structures A1 and B1, which are calculated to be the lowest in energy of each pair, will be included in the discussion.

As displayed in Figure 3, the hydrogen bond lengths differ between structures A1 and B1. In Structure A1, there are three hydrogen bonds with bond lengths of 1.68, 1.84, and 1.82 Å, of which the latter two refer to the hydrogen bonds that share the same acceptor atom. In structure B1, there are three hydrogen bonds as well, with bond lengths of 1.84 Å, 1.54 Å, and 1.54 Å. The length is correlated to the strength of the H-bonds, with stronger H-bonds being shorter.^65^

In Table 1, the relative energies of the A1 and B1 structures, calculated using different methods, are shown. For the B3LYP-D3­(BJ), the MP2, and the revDSD-PBEP86-D4 calculations, the geometries have been optimized at the respective levels of theory with very tight cutoffs, followed by calculation of the harmonic vibrational frequencies and harmonic zero-point energies. The CBS-QB3 method has its own predefined internal workflow. CCSD­(T) energies are calculated at the MP2 optimized geometry, and MP2 harmonic zero-point energies are used. D_*e*_ and D_0_ values are calculated with respect to monomeric units calculated at the same levels of theory.

**Table 1:** Electronic Energies (*E*), Dimer Binding Energies (*D*_e_), and Harmonic Zero-Point Energy Corrected Binding Energies (*D*_0_) Together with Their Relative Values for Structures A1 and B1 at Different Levels of Theory^a^

		Structure A1	Structure B1	Δ[A1–B1]
B3LYP-D3(BJ)	*E* (Hartree)	–1288.28412	–1288.28394	
	*D*_e_ (kJ/mol)	194.51	194.04	0.47
	*D*_0_ (kJ/mol)	185.70 (186.98)	188.83 (188.69)	–3.13 (−1.71)
MP2	*E* (Hartree)	–1286.28583	–1286.28522	
	*D*_e_ (kJ/mol)	192.37	190.76	1.61
	*D*_0_ (kJ/mol)	183.69 (184.97)	185.82 (185.65)	–2.13 (−0.68)
revDSD-PBEP86-D4	*E* (Hartree)	–1286.75435	–1286.75317	
	*D*_e_ (kJ/mol)	189.59	186.49	3.10
	*D*_0_ (kJ/mol)	180.41 (181.79)	180.82 (180.87)	–0.41 (0.92)
CBS-QB3	*E* (Hartree)	–1286.52029	–1286.51889	
	*D*_e_ (kJ/mol)	196.81	193.12	3.69
	*D*_0_ (kJ/mol)	187.97 (189.26)	188.01 (187.96)	–0.04 (1.30)
CCSD(T)^a^	*E* ^a^ (Hartree)	–1286.37765	–1286.37618	
	*D*_e_ ^a^ (kJ/mol)	193.74	189.89	3.85
	*D*_0_ ^b^ (kJ/mol)	185.06 (186.33)	184.94 (184.78)	0.12 (1.55)

a *D*_0_ for the deuterated species are provided in brackets. All calculations (except CBS-QB3) are performed using the aug-cc-pV­(T+d)­Z basis set.

b Single point CCSD­(T) energies at MP2 geometries.

c Using MP2 zero-point energies.

For all five methods, the energy difference between the two structures is small and on the order of the uncertainty of the methods. When considering just the electronic energy, all methods predict the A1 structure to be lower in energy; however, when including harmonic zero-point energies, the difference between the energy of the two structures decreases, and for some of the methods, the preference shifts toward B1. Probably the most reliable energetics come from the CCSD­(T) calculations, which predict the A1 structure to be lower in energy based on both the electronic energies (D_*e*_) and when the zero-point energy is included (D_0_). It should be noted that the CBS-QB3 values are close to the CCSD­(T) values; however, they were obtained at a fraction of the computational costs of the CCSD­(T) calculations.

With the A1 and B1 isomers being so close in energy, an important question is how those two structures might interconvert. Given the large structural differences, it seems that for interconversion, many H-bonds have to be broken and reformed, concomitant with large movements of heavy atoms. It therefore seems that a pathway would involve barriers that are not likely to be surmounted under our experimental conditions and probably also not at room temperature.

For structures A and B, an anionic dihydrogen phosphate unit is bound to a neutral phosphoric acid unit, and an important process, also relevant for dynamics that might occur in the condensed phase, is the transfer of protons between the two units. A facile exchange would occur via a transfer of a hydrogen-bonded proton. In the case of structure A1, all three protons involved in hydrogen bonding are located on the neutral phosphoric acid unit, and a transfer of any of them would therefore seem possible. However, both rigid and relaxed potential energy scans along the corresponding transfer coordinate are purely uphill with no local minimum along the proton transfer coordinate.

For structure B, the proton involved in the upper H-bond (see Figure 3) is already located on the anionic moiety and would therefore not transfer. However, this is not the case for the two protons involved in the lower H-bonds. A (relaxed) potential energy scan along one of the proton transfer coordinates leads to B1 or B2, where the acid-conjugate base roles are switched. The barrier for such a transfer is calculated to be only ∼500 cm^–1^ (see Figure S5) and most likely lower than the corresponding zero point energy in that coordinate. Structure B is therefore expected to have a highly dynamic structure with delocalized protons.

Infrared spectra for both the A1 and B1 structures, as well as the monomeric units, are calculated within the harmonic approximation using the B3LYP-D3­(BJ), MP2, and revDSD-PBEP86-D4 methods. As can be seen in Figures S7 and S8, the differences among the resulting IR spectra are small. The B3LYP-D3­(BJ) calculation requires the least computational effort, and it has been proven in the past to provide reliable predictions for IR spectra of gas-phase ions. Further, due to its limited computational cost, the use of B3LYP-D3­(BJ) allows us to go beyond the harmonic approximation. Therefore, we focus on this method for the comparison to the experimental results.

In the experimental spectrum of H_2_PO_4_^–^ (Figure 1, Figure S11) in the mid-IR region, six bands at 775, 800, 1034, 1046, 1105 and 1315 cm^–1^ are observed. Calculating the harmonic frequencies using B3LYP-D3­(BJ)/aug-cc-pVTZ (MP2 results in brackets), we obtain the following unscaled harmonic frequencies: 746 (765), 773 (794), 1040 (1042), 1057 (1060), 1077 (1084), and 1297 (1305) cm^–1^. The agreement between the B3LYP-D3­(BJ) and MP2 results is therefore good. The first two modes can be assigned to symmetric ν_*s*_ and antisymmetric ν_*as*_(HO–P–OH) stretching modes, followed by two δ­(P–O–H) bending vibrations, while the last two stem from the symmetric ν_*s*_ and antisymmetric ν_*as*_(O–P–O^–^) stretching modes of the POO^–^ group. The δ­(P–O–H) bending modes are therefore calculated to be 6–14 cm^–1^ higher in frequency than observed experimentally. This is expected, as they do not include anharmonicity effects, and including those by perturbative treatment or an empirical scaling factor would shift them to lower wavenumbers, in the right direction. The situation is, however, different for the ν­(O–P–O^–^) and the ν­(HO–P–OH) stretching modes. Here, theory is 10–29 cm^–1^ lower in frequency than experiment. Including corrections for anharmonicities would make the situation even worse.

It has been reported that for an accurate description of bonding involving second row elements, an additional set of tight d-functions is important.^66,67^ We therefore performed the same calculations using the aug-cc-pV­(T+d)­Z basis set^66^ downloaded from the basis set exchange.^68^ The frequencies using the more flexible basis set for the modes described above are now: 757 (773), 781 (800), 1040 (1042), 1058 (1060), 1091 (1094), and 1312 (1316) cm^–1^ (see Figure S11). The inclusion of the additional d-function of the phosphorus atoms thus does not affect the location of the δ­(P–O–H) bending modes; however, it shifts the ν­(O–P–O^–^) stretching modes 6–14 cm^–1^ to higher frequencies. While this is a shift in the right direction, an accurate description should end up with harmonic values that are 1–2% higher than the experiment. This is not the case, and we conclude that the aug-cc-pV­(T+d)­Z basis set gives significantly better results, compared to the aug-cc-pVTZ basis set, but there seems to be room for improvement.

### Comparison between Experiment and Theory

In the case of noninteracting phosphoric acid and dihydrogen phosphate anion units, the resulting IR spectrum would be the sum of the IR spectra of both components. Figure 4 (and for the deuterated species, Figure S6) shows the experimental spectrum of dPAD-H_5_ in the fingerprint region compared to the calculated spectra of H_2_PO_4_^–^ and H_3_PO_4_.

**Figure 4:**
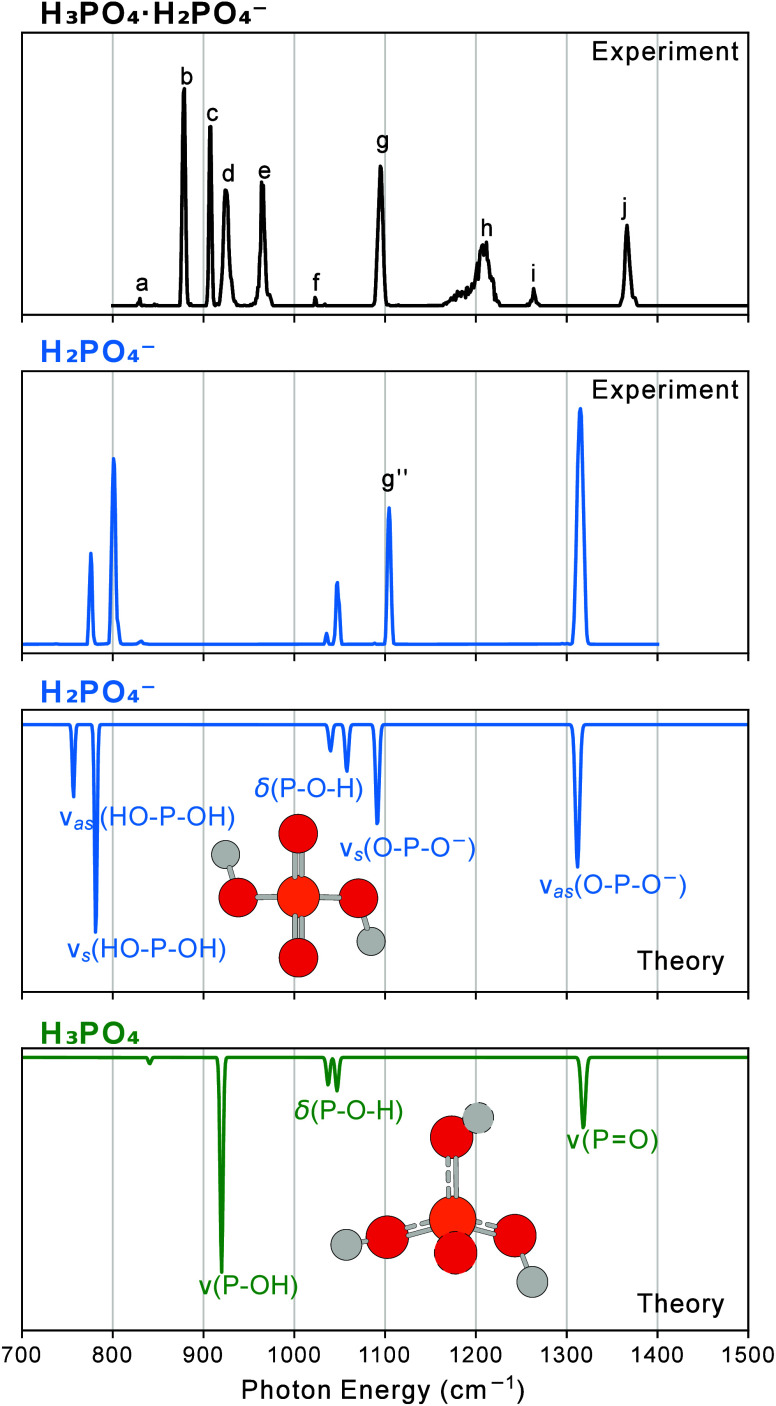
Cryogenic IR action spectrum, recorded using helium nanodroplets, of the deprotonated phosphoric acid dimer dPAD-H_5_ (upper panel) compared to the calculated unscaled IR spectra of the neutral phosphoric acid (H_3_PO_4_) and dihydrogen phosphate (H_2_PO_4_^–^) anion, at the B3LYP-D3­(BJ)/aug-cc-pV­(T+d)­Z level of theory in the harmonic approximation.

The IR-spectrum calculated for H_2_PO_4_^–^ in the wavenumber range shown in Figure 4 has been described in the previous section and is dominated by the ν_*s*_ and ν_*as*_(O–P–O^–^) stretching modes at 1091 and 1312 cm^–1^ and the δ­(P–O–H) bending modes at 1040 and 1058 cm^–1^. Predicted strong HO–P–OH stretching modes are located below 800 cm^–1^. Upon deuteration, the δ­(P–O–D) bending modes shift to 803 and 833 cm^–1^.

H_3_PO_4_ is calculated to be of *C*_*3*_ symmetry, and the most intense band in the spectrum stems from ν­(P—OH) and ν­(P=O) stretching vibrations. The bands in the calculated spectrum that are assigned to the ν­(P—OH) stretching modes are the peak with low intensity at 841 cm^–1^, which is assigned to the excitation of the symmetric ν_*s*_(P—OH) stretch, and a strong transition at 920 cm^–1^, which reflects a transition involving the doubly degenerate ν­(P—OH) stretch. The ν­(P=O) mode is located at 1318 cm^–1^. The two bands in between stem from δ­(P—O—H) bending vibrations for which one peak is located at 1037 cm^–1^ and a doubly degenerate mode is at 1047 cm^–1^. It can be observed that the band calculated for the symmetric stretching mode of the ν_*s*_(POO^–^) group is located close in position to band (g) in the experimental spectrum. However, there is no clear experimental counterpart of the antisymmetric ν_*as*_(O—P—O^–^) stretching mode in the experimental spectrum, nor of the ν­(P=O) stretching mode, which are both predicted to be very close to each other.

In Figure 5, the experimental IR action spectra of dPAD-H_5_ and dPAD-D_5_ in the fingerprint region are compared with the spectra calculated in the harmonic approximation for structures A1 and B1. For dPAD-H_5_ in the region below 1000 cm^–1^, four intense bands (b–e) are observed experimentally, while only three intense bands are predicted in this region for A1. Above 1000 cm^–1^, four bands (g–j) are observed, of which (g) and (j) are highly diagnostic and have clear counterparts in the calculated spectrum of A1. The strong transition near 1100 cm^–1^ (g) matches in frequency with the predicted symmetric stretching vibration of the POO^–^ group (ν_*s*_(O—P—O^–^)). The predicted mode which corresponds to band (j) has mainly ν­(P=O) stretching character, mixed with some δ­(P—O—H) bending motion of the —OH group on H_3_PO_4_, which is singly coordinated to the POO^–^ group of the H_2_PO_4_^–^ unit (the upper OH group in Figure 3). Another characteristic feature is that peak (h) is the only peak that is unusually broad. A similar broadening was observed in another phosphoric acid-containing system, the phosphoric acid–formate dimer (FP), studied by the same IR action technique, and which has a similar hydrogen bonding motif.^40^ In both cases, the frequency of this broad peak is close in frequency to that of the predicted bending mode of the two weakly bound hydrogen atoms sharing the double acceptor (1165 cm^–1^ in the calculated spectrum of A1; this mode is absent in structure type B). In the FP investigation, the broadening was attributed to two possible origins: anharmonic couplings or interaction with the helium environment.^40^

**Figure 5:**
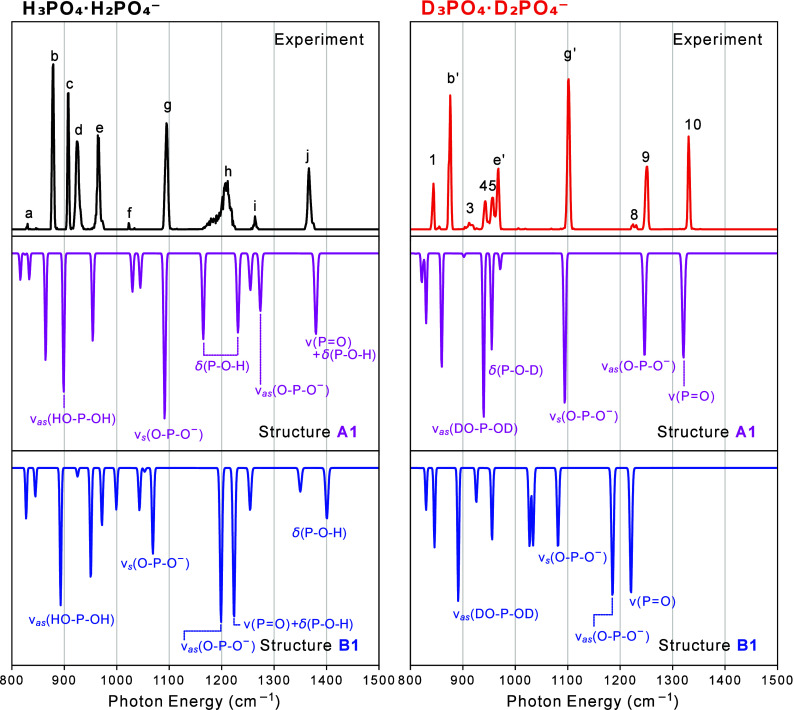
Cryogenic IR action spectra, recorded using helium nanodroplets, of the deprotonated phosphoric acid dimer dPAD-H_5_ (left panel) and its fully deuterated counterpart dPAD-D_5_ (right panel) are compared with the unscaled calculated IR action spectra of structures A1 (magenta) and B1 (blue) at the B3LYP-D3­(BJ)/aug-cc-pV­(T+d)­Z level of theory in the harmonic approximation. The most prominent modes of the calculated spectra are labeled in the figure. A complete description of the vibrational modes is included in the Supporting Information.

Despite this overall agreement, some discrepancies remain. In particular, the presence of more experimental peaks in the region below 1000 cm^–1^ than those predicted for structure A1 alone.

When comparing the experimental spectrum of dPAD-H_5_ with the calculated spectrum of structure B1, we can notice that only a few experimental bands have counterparts (e.g., (c), (e), and (h)); however, most do not.

The experimental spectrum of dPAD-D_5_ in the mid-IR range agrees well with the calculated spectrum of structure A1, and all experimental bands have calculated counterparts. The peaks calculated to be between 800 and 1000 cm^–1^ have mostly ν­(P-OD) stretch mixed with δ­(P—O—D) bend character. For dPAD-D_5_, only three modes have calculated frequencies between 1000 and 1500 cm^–1^ (with the next higher frequency modes being the O—D stretching modes). They are located at 1094 cm^–1^ (ν_*s*_(O—P—O^–^)), 1247 cm^–1^ (ν_*as*_(O—P—O^–^)) and 1321 cm^–1^ (ν­(P=O)). The assignments in brackets indicate the main contributions to these normal modes. A detailed assignment can be found in Table S2, in the Supporting Information. In contrast, the calculated spectrum of structure B1 predicts no transitions above 1250 cm^–1^, where two intense peaks are observed experimentally. The calculated spectrum for structure B1 shows a poor agreement with no clear correlation to the experimental spectrum of dPAD-D_5_.

In the case of dPAD-H_5_, the agreement of the experimental spectrum with the calculated spectrum for structure A1 is much better than that of structure B1. Nonetheless, the agreement is also not perfect, which makes it difficult to do a firm assignment based on dPAD-H_5_ data, whether A1, B1, or a mix of the two, is present. However, for dPAD-D_5_ the situation seems clear, and the excellent agreement of the experimental spectrum to the calculated spectrum for structure A1 allows us to assign dPAD-D_5_ to structure A1. The poorer agreement between experiment and calculation for dPAD-H_5_ most likely reflects the fact that the OH wagging (δ­(P–O–H)) and ν­(O–P–O^–^) stretching vibrations have similar frequencies. This leads to normal modes that are more mixed in character than in dPAD-D_5_. For this reason, slight differences in the accuracy of the treatment of these two types of vibrations will be more notable in the calculated spectrum for dPAD-H_5_ than for dPAD-D_5_.

In Figures 1 and 5, three peaks ((e) and (e′), (b) and (b′) as well as (g) and (g′)), are at nearly the same position in the spectra of dPAD-H_5_ and dPAD-D_5_. Band (g) is also found in the spectrum of H_2_PO_4_^–^ and labeled (g″). Theory identifies all three of the (g) peaks as arising from the ν_*s*_(O—P—O^–^) symmetric stretch, and predicts their positions well. This is not the case for the peaks labeled (b) and (e), where the closest calculated peaks involve light atom motions for which isotopic shifts occur. Instead, additional modes that are responsible for peaks in the calculated spectra that shows isotopic shifts are the antisymmetric ν_*as*_(POO^–^) and the ν­(P=O) stretching modes, which can be assigned to bands (i) and (9) and to bands (j) and (10) in the spectra of dPAD-H_5_ and dPAD-D_5_, respectively. An interesting aspect is that, typically, harmonic calculations require a scaling factor (<1) to correct the overestimation of the frequencies.^69^ However, as illustrated in Figure 5, some band positions are already predicted to be at lower frequencies than the experimentally observed bands. This effect has been observed in other investigations of phosphate-containing molecules.^40,43^ In the case of the dihydrogen phosphate anion, we also observe this trend when calculating the frequencies at different levels of theory (see Figure S11).

Figure 6 compares the experimental spectra of dPAD-H_5_ and dPAD-D_5_ in the O–H (O–D)-stretching region with calculated harmonic as well as VPT2 spectra evaluated for structures A1 and B1. Spectra in the harmonic approximation predict for both complexes for A1 and B1, a weak band at the high frequency stemming from stretching motion of the H­(D) atoms, which are not involved in H-bonding, and red-shifted bands stemming from H­(D) atoms involved in H-bonding. However, for the harmonic spectra, neither the number of calculated strong bands nor their positions reproduce the experimental spectra. Calculated spectra that include the effects of anharmonicities via VPT2 are shown as black traces. Their inclusion drastically alters the calculated spectra by shifting fundamental bands, by altering their intensities, and by adding combination bands. For dPAD-H_5_, the VPT2 spectrum of A1 now shows three prominent bands between 2800 and 3400 cm^–1^, in qualitative agreement with the experiment. In the same region, the VPT2 spectrum of B1 shows only one prominent and one weak band. In both calculated spectra, a weaker band near 3600 cm^–1^ is predicted. For dPAD-D_5_, the VPT2 spectrum of A1 shows three strong bands between 2100 and 2600 cm^–1^ and a weaker band near 2700 cm^–1^. For B1 between 2100 and 2600 cm^–1^, only two stronger bands are present, accompanied by a weaker band near 2700 cm^–1^. As will be shown later, those weaker bands near 3600 cm^–1^ (dPAD-H_5_) and near 2700 cm^–1^ (dPAD-D_5_) correspond to sharp bands in the experiment, so that their integrated intensities are in rough agreement with experiment. Based on this comparison, we can conclude that the A1 spectra provide a much better match to the experiment, and the B1 structure will not be discussed further.

**Figure 6:**
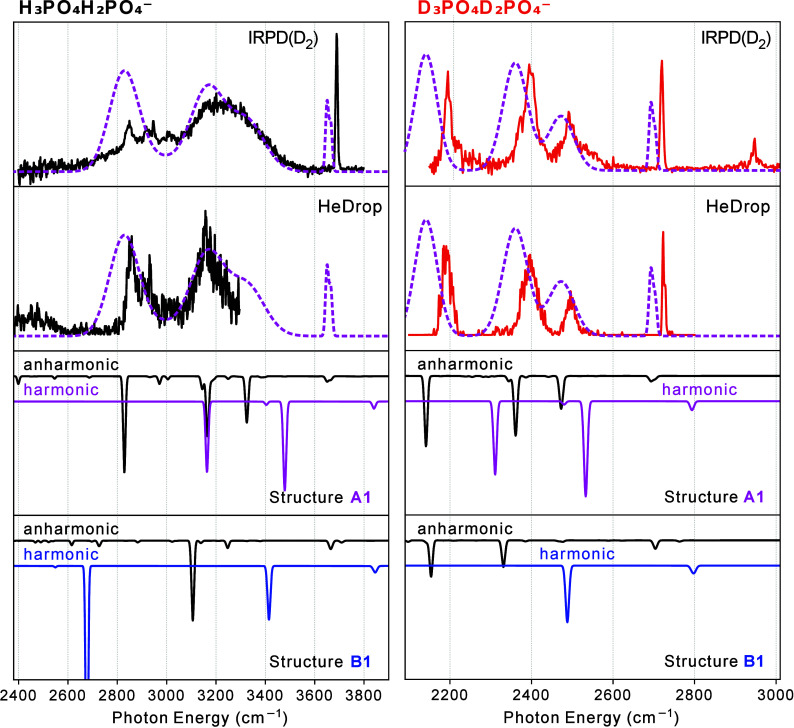
IR Photodissociation (IRPD) spectra using D_2_ tagging and spectra recorded using Helium Nanodroplets (HeDrop) of the deprotonated phosphoric acid dimer dPAD-H_5_ (left panel) and its fully deuterated counterpart dPAD-D_5_ (right panel) compared to the unscaled calculated IR action spectra of structures A1 (magenta) and B1 (blue) at the B3LYP-D3­(BJ)/aug-cc-pV­(T+d)­Z level of theory in the harmonic approximation. Spectra calculated in anharmonic approximation using VPT2 are shown in black lines. The magenta dashed lines overlaying the experimental spectra result from convoluting the VPT2 spectra of A1 with broad Gaussian functions for the lower frequency modes and narrow Gaussian functions for the high frequency modes (see text).

For dPAD-H_5_, the five H atoms in the complex lead to five fundamental bands in the O–H stretching region. The free −OH groups lead to two weak bands at the high-frequency side of the spectrum. OH stretching motion of the H atom that is singly coordinated to a O atom of the POO^–^ group of the H_2_PO_4_^–^ unit (the upper one in structure A1, shown in Figure 3) leads to the most intense band at the low frequency end in the calculated spectrum in Figure 6 (for both, the harmonic and the anharmonic case). The inclusion of anharmonicities adds six combination bands with an intensity of ≥2% of that of the most intense band. In all cases, these states involve one quantum of excitation in an OH stretching vibration and one in a low-frequency vibration that modulates the hydrogen bond strength either by changing the O–O distances, as is the case for modes 5 and 11, or through a rocking vibration of the two OH bonds, which form hydrogen bonds to the same oxygen atom. Similar combination transitions have been seen in ion–water or similar complexes, and their intensity can be rationalized through an adiabatic separation of the high and low frequency vibrations.^70–72^

In the experiment, the structure below 3600 cm^–1^ is broad, and only the IRPD band at 3690 cm^–1^ is narrow. The reason appears to be homogeneous broadening due to couplings between the hydrogen-bonded OH stretching vibrations to low-frequency torsional modes in the complex, which break the hydrogen bonds. Similar behavior has been seen in the IRPD spectra of other strongly hydrogen-bonded systems, for example, the complex of H_3_O^+^ with 18-crown-6 ether.^72^ As in the present study, a progression of resolvable peaks is seen in the OD stretching region while a single broad peak is found in the OH stretching region. In the earlier work, these features were modeled by considering transitions to states with one quantum of excitation in the OH or OD stretch and several quanta of excitation in low-frequency vibrations that break the hydrogen bond. In the present study, we use perturbation theory, which only includes transitions with up to two quanta of excitation. Consequently, only the first two peaks in this progression are included in the calculated spectrum. The longer progressions are modeled by the dashed lines in Figure 6, which show the spectrum that is obtained for dPAD-H_5_ when the calculated anharmonic transitions for the two modes of the free −OH groups are convoluted with 0.4% fwhm Gaussian functions, while all other modes are convoluted with 5% Gaussians. Clearly, the agreement with experiment is very good; however, the band from the free −OH groups still appears broader and less intense, compared to the experiment. The broadening of this peak reflects a 1:1 coupling between these two OH stretching vibrations in the VPT2 calculation, which is nonzero due to the calculated low symmetry of the ion. When zero-point effects are properly included, dPAD is expected to have C_*s*_ symmetry, and this coupling term vanishes.

Shown on the right side of Figure 6 are the spectra for dPAD-D_5_. When analyzing the results from the anharmonic calculations, a similar picture as for dPAD-H_5_ emerges. Again, five fundamental bands are present, which shift and change intensities upon inclusion of anharmonicities. The band stemming from the free −OD groups is the most to the blue, while the one from the singly coordinated OD group is the most intense and the most red-shifted. For dPAD-D_5_ in this spectral range, only two combination bands with an intensity of ≥2% of that of the most intense band appear. The most intense of those is located at 2473 cm^–1^ and it is the third most intense band in the overall spectrum. As in the case of dPAD-H_5_, this band involves a fundamental −OD stretch mode of the two lower −OD groups (Figure 3) combined with a low-frequency torsional mode. Shown with dashed lines is a convolution of the calculated anharmonic spectrum, again with 0.4% Gaussian function for the free-OD modes, but this time 3% for all other modes. Clearly, the agreement to the experiment is very good, and the combination band can be clearly identified in the experimental spectrum.

In the Supporting Information (Figure S9), the mid-IR spectra at the VPT2 level of dPAD-H_5_ and dPAD-D_5_ for structure A1 are shown. The changes when comparing harmonic and VPT2 are not as large as in the O–H and O–D stretching regions, but they are still significant. For both dPAD-H_5_ and dPAD-D_5_, fundamental modes lose significant amounts of intensity, and a large number of combination bands with weak to medium intensity appear. Especially in the case of dPAD-D_5_, this is not what is observed in the experiment, and the agreement between experiment and theory is better for the spectra at the harmonic approximation, compared to the anharmonic calculations. The introductions of the additional bands from the VPT2 calculation that are not observed in experiment most likely reflect the limitations of VPT2. In this spectral region, the combination of the relatively large number of two and three-quanta states reflects excitation of anharmonic vibrational modes that involve displacements of many of the atoms, making it difficult to identify a small resonance space that removes the near-degeneracies that plague perturbation theory calculations.

One important finding of this study is that IRPD spectra of isolated D_2_ tagged species are very similar to IR spectra recorded by using the helium droplet technique. Further, spectra obtained using both techniques indicate that the deprotonated phosphoric acid dimer complex adopts structure A1 (see Figure 3). In the literature, structures for protonated,^44^ neutral,^41,43,45^ deprotonated^33^ and doubly deprotonated^42^ phosphoric acid dimers have been proposed. None of those structures for cationic and neutral clusters resembles structure A1; however, some neutral clusters lead to B1 when removing one proton. In the calculations on the anionic dimer,^33^ a structure similar to B1 is identified as the lowest energy structure. Some of the structures calculated for the dianion^42^ show some resemblance with A1 as they also feature coordination of two −OH groups to one O atom, but they are otherwise different. However, A1 is very similar to the structural motif found for the phosphoric acid-formate anionic dimer.^40^ It is therefore possible that the hydrogen bonding motif in A1 is general for phosphoric acid.

## Conclusions

In this work, we investigated dPAD-H_5_ and its deuterated analogue using cryogenic IR action spectroscopy in helium nanodroplets and IRPD spectroscopy of the corresponding D_2_-tagged species. Our results show that the two lowest-energy structures (A1 and B1) are nearly isoenergetic, highlighting the limitations of theory-based predictions alone for structural assignment. However, spectroscopic evidence obtained by both experimental techniques strongly supports an assignment to the previously unreported structure A1, where the proton is shared across a network of three hydrogen bonds involving five oxygen atoms. In structure A1, the two oxygen atoms of the phosphate group function as single and double hydrogen bond acceptors, while the protons of phosphoric acid act as donors (AA2/DDD). Other studies on phosphate-containing compounds report a similar coordination of two −OH groups to a single oxygen atom,^40,42^ suggesting that the hydrogen-bonding motif in A1 is likely common for such systems. This system can serve as a model for understanding the role of phosphoric acid in proton transfer processes within ionic hydrogen-bonded complexes. Further experiments investigating larger deprotonated phosphoric acid clusters could enhance our understanding of the role of anions in proton transfer dynamics.

## Supplementary Material



## Notes

Additional structures of the deprotonated dimer of phosphoric acid, the Cartesian coordinates of the species investigated here, as well as the results of energy and frequency calculations at different levels of theory (PDF)
