# Supplementary material for: Cryogenic Vibrational Spectroscopy of the Deprotonated Dimer of Phosphoric Acid
Source: J Phys Chem A. 2025 Dec 15;130(5):993–1005. doi: 10.1021/acs.jpca.5c06704 (PMC12884523; doi:10.1021/acs.jpca.5c06704)
Supplement: Supplementary file 1 [file jp5c06704_si_001.pdf]

Cryogenic Vibrational Spectroscopy of the Deprotonated Dimer of Phosphoric Acid: Supplementary Information

América Y. Torres Boy,† Jia Han,‡ Gurpur Rakesh D. Prabhu,†,¶ Martín I. Taccone,† Anoushka Ghosh,† Hannah Buttkus,‡ Katja Ober,† Gerard Meijer,† Knut R. Asmis,‡ Anne B. McCoy,§ and Gert von Helden*,†

†*Fritz Haber Institute of the Max Planck Society, 14195 Berlin, Germany*

‡*Wilhelm-Ostwald-Institut für Physikalische und Theoretische Chemie, Universität Leipzig, Linnéstraße 2, 04103 Leipzig, Germany*

¶*Institute of Chemistry and Biochemistry, Freie Universität Berlin, 14195 Berlin, Germany*

§*Department of Chemistry, University of Washington, Seattle, 98195, WA, USA*

E-mail: helden@fhi.mpg.de

Contents

1. **Spectrum of $H_2PO_4^-$ in the OH stretching region** S3
2. **Calculated structures and energies of the deprotonated dimer of phosphoric acid** S4
3. **List of vibrational transitions** S9
4. **Mid IR spectra of the deuterated deprotonated dimer of phosphoric acid compared to calculations of the individual components** S12
5. **Mid IR spectra of the deprotonated dimer of phosphoric acid in comparison of calculations at different levels of theory** S13
6. **Spectra of the deprotonated dimer of phosphoric acid at the VPT2 level** S15
7. **Spectrum of $H_2PO_4^-$ and the effect of anharmonicity** S17
8. **Cartesian coordinates of the optimized structures** S19

1. Spectrum of $H_2PO_4^-$ in the OH stretching region

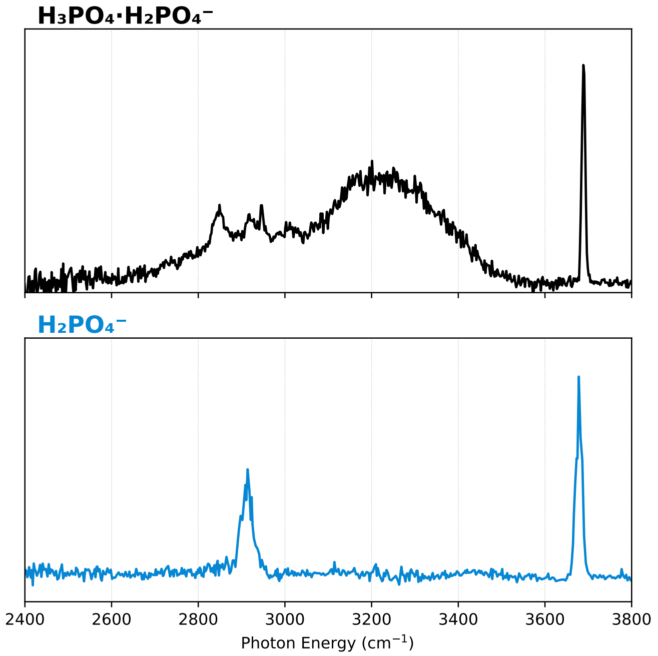

Figure S 1: Comparison of IR Photodissociation (IRPD) spectra of the deprotonated dimer of phosphoric acid dPAD-H_5 (top panel), and the dihydrogen phosphate ion (bottom panel).

The spectra of the deprotonated dimer of phosphoric acid in the OH stretching region are recorded using an IR photodissociation spectroscopy (IRPD). The OH stretching bands are found to be located between 2800 and 3750 cm^{-1}. In the region between 2800 and 3300 cm^{-1}, at least three broad features can be identified. Remarkably, a band located at $\approx$3700 cm^{-1} is drastically sharper. When comparing with the IRPD spectrum of the dihydrogen phosphate anion (Fig. S1), the presence of a similarly sharp line at around the same frequency in the spectra of dPAD-H_5 and dihydrogen phosphate suggests that this band can be associated with the OH stretching vibrations of hydroxyl groups not involved in hydrogen bonding.

2. Calculated structures and energies of the deprotonated dimer of phosphoric acid

When performing a search through the structural landscape, two low-energy structure types are found: A and B. Both structure types are composed of two distinct moieties: phosphoric acid and dihydrogen phosphate, which are connected via hydrogen bonding. In both structure types, the negative charge is delocalized among the two non-protonated oxygens of dihydrogen phosphate.

In A, the three hydrogen atoms of phosphoric acid point towards the POO^- group of dihydrogen phosphate. The two -OH groups of dihydrogen phosphate are not involved in H-bond interaction. Interestingly, only five oxygen atoms participate in the three H-bonds. In contrast, type B shows a bonding motif consisting of three hydrogen bonds involving six oxygen atoms, three from phosphoric acid and three from dihydrogen phosphate. This structure type also shows two "external" -OH groups, which do not participate in hydrogen bonding.

Structure types A and B each have two structurally very similar conformers, distinguished by the orientation of the non-bonding OH groups (see Fig. S2, Table S1). In structure type A, those conformers differ by trans (A1) or cis (A2) orientation of the OH groups, and in type B, the OH group on the formally neutral unit differs in orientation by $\sim 180°$. The heavy atom positions and bond lengths in these analogous structures are similar, and so are their calculated IR spectra (Fig. S3, S4). The Cartesian coordinates of the optimized structures are shown in Section 8 of this document.

Table S 1: Electronic energies (E), dimer binding energy (D_e), and zero-point corrected (D_0) as well as their relative values are shown for structures A1, A2, B1, and B2 calculated at the B3LYP-D3(BJ)/aug- cc-pV(T+d)Z level of theory. The values of the D_0 of the deuterated species are provided in brackets. All the energies in the table, except electronic energies (E), are displayed in kJ/mol.

Structure	A1	A2	B1	B2
E (Hartrees)	-1288.28412	-1288.28363	-1288.28394	-1288.28386
D_e	194.51	193.24	194.04	193.82
D_0	185.70 (186.98)	184.52(185.77)	188.83(188.69)	188.54(188.34)
relative D_e	0.00	-1.27	-0.47	-0.69
relative D_0	-3.13(-1.71)	-4.31(-2.92)	0.00(0.00)	-0.29(-0.35)

* Electronic energies of the lowest energy structure of $H_2PO_4^-$ and H_3PO_4 were calculated to be -643.84 and -644.37 Hartrees, respectively.

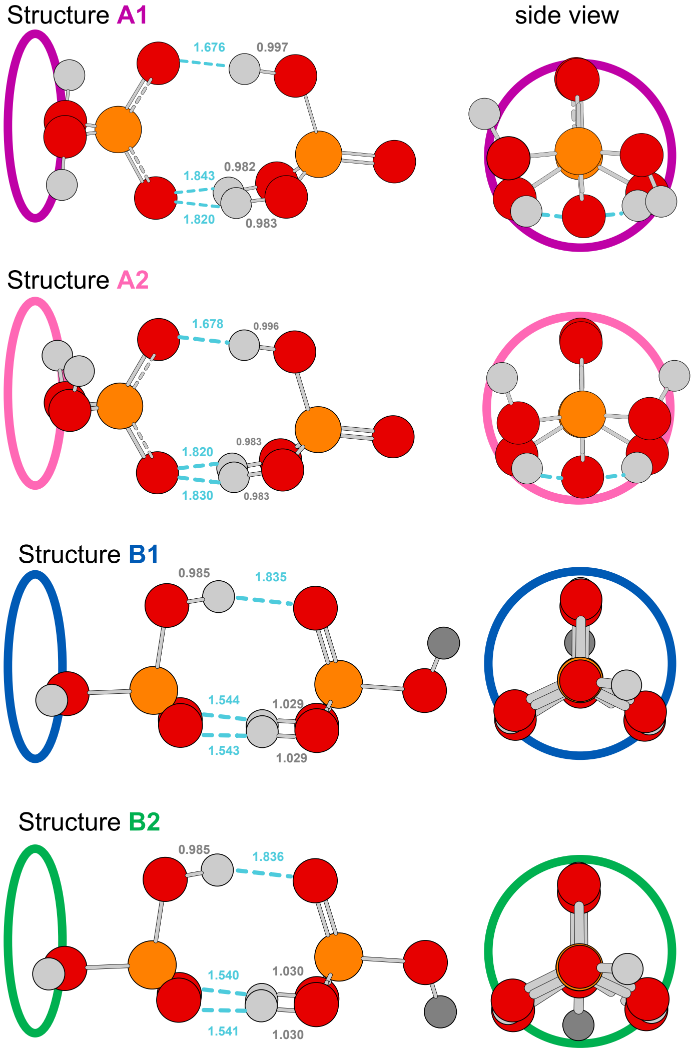

Figure S 2: Graphic representations of the four most relevant optimized structures: A1, A2, B1 and B2. The lengths (Å) of the bonds involved in the hydrogen bonding are shown.

The calculated spectra for structures A1 and A2 are almost identical. On the other hand, the differences among the spectra for structures B1 and B2 are greater, especially in the region between 950 and 1280 cm^{-1}. This is due to the different couplings of PO stretching modes to the bending motions of the differentiating hydrogen atom.

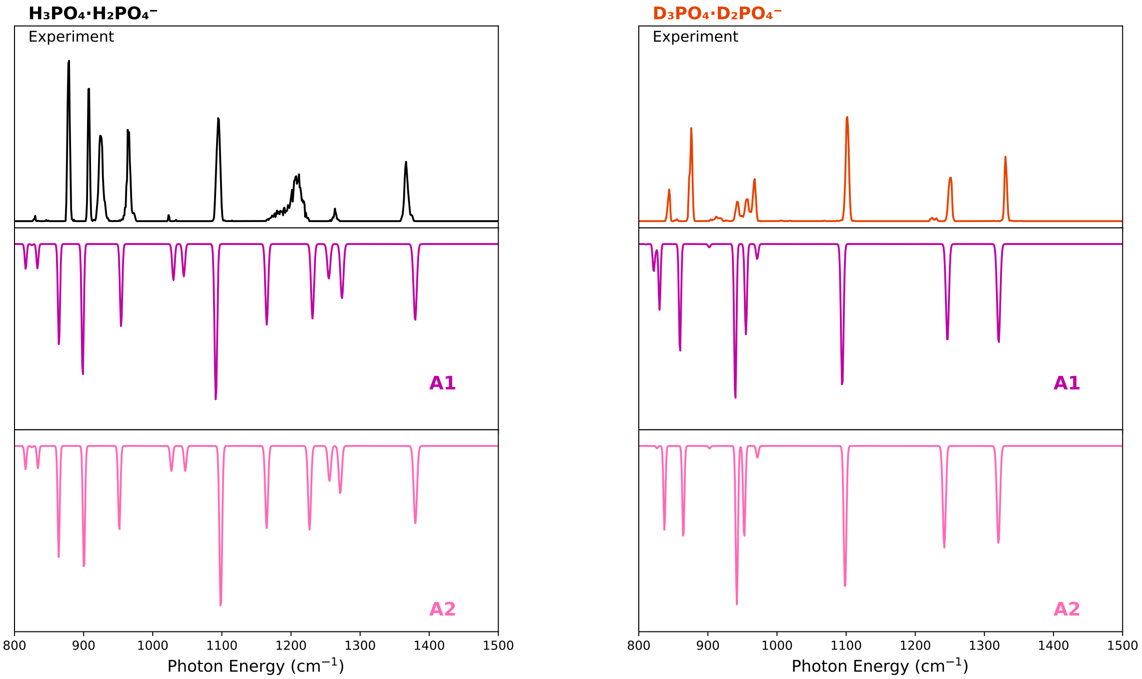

Figure S 3: IR action spectra recorded using helium nanodroplets of the deprotonated dimer of phosphoric acid dPAD-H_5 (left panel) and its fully deuterated counterpart dPAD-D_5 (right panel) compared to the calculated IR spectra of structure A1 (magenta), A2 (pink) at the B3LYP-D3(BJ)/aug- cc-pV(T+d)Z level of theory in the harmonic approximation. No scaling factor is used.

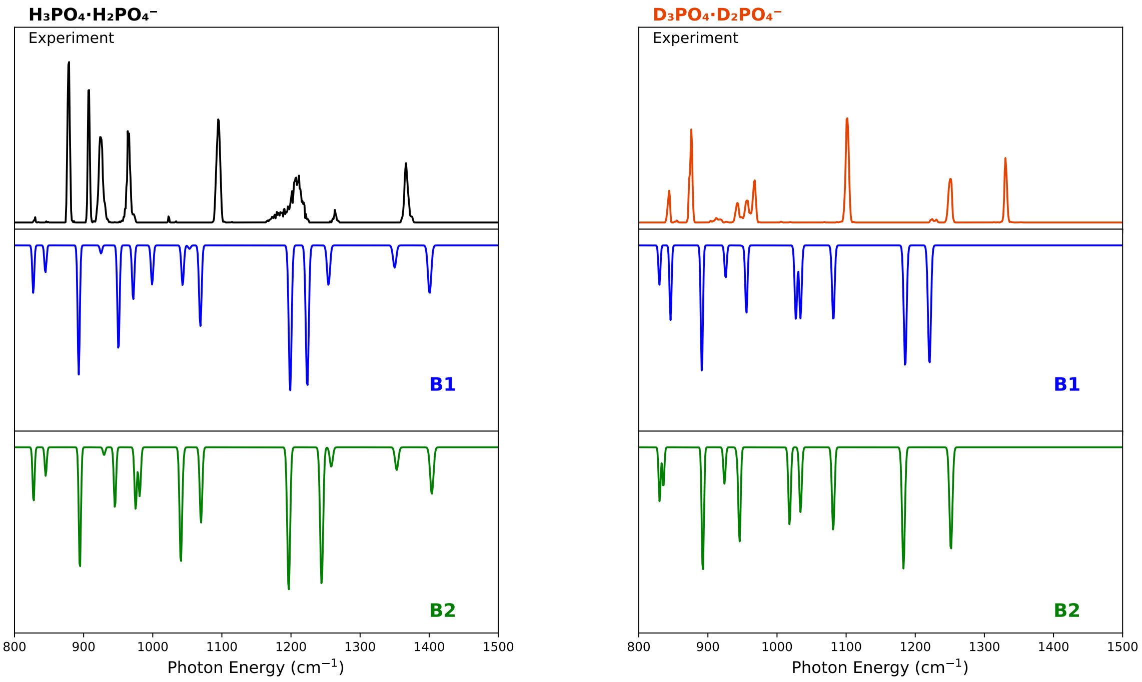

Figure S 4: IR action spectra recorded using helium nanodroplets of the deprotonated dimer of phosphoric acid dPAD-H_5 (left panel) and its fully deuterated counterpart dPAD-D_5 (right panel) compared to the calculated IR spectra of structure B1 (blue) and B2 (green) at the B3LYP-D3(BJ)/aug- cc-pV(T+d)Z level of theory in the harmonic approximation. No scaling factor is used.

Visual inspection shows that although A1 and B1 are calculated to be nearly isoenergetic, they are structurally very different, and no clear low-energy coordinate linking the two can be identified. The interconversion between A1 and A2 involves a barrier associated exclusively with the cis–trans reorientation of the non-bonding OH groups. In contrast, the isomerization between B1 and B2 (and vice versa) can be mediated by either the rotation of a non-bonding OH group or a second coordinate involving proton transfer of one of the two lower H-bonded hydrogen atoms. This transfer leads directly to another minimum-energy structure in which the acid–base roles are switched (see Fig. S5). The barrier calculated of $\approx$500 cm^{-1} is much lower than the expected vibrational frequency of the OH stretching mode.

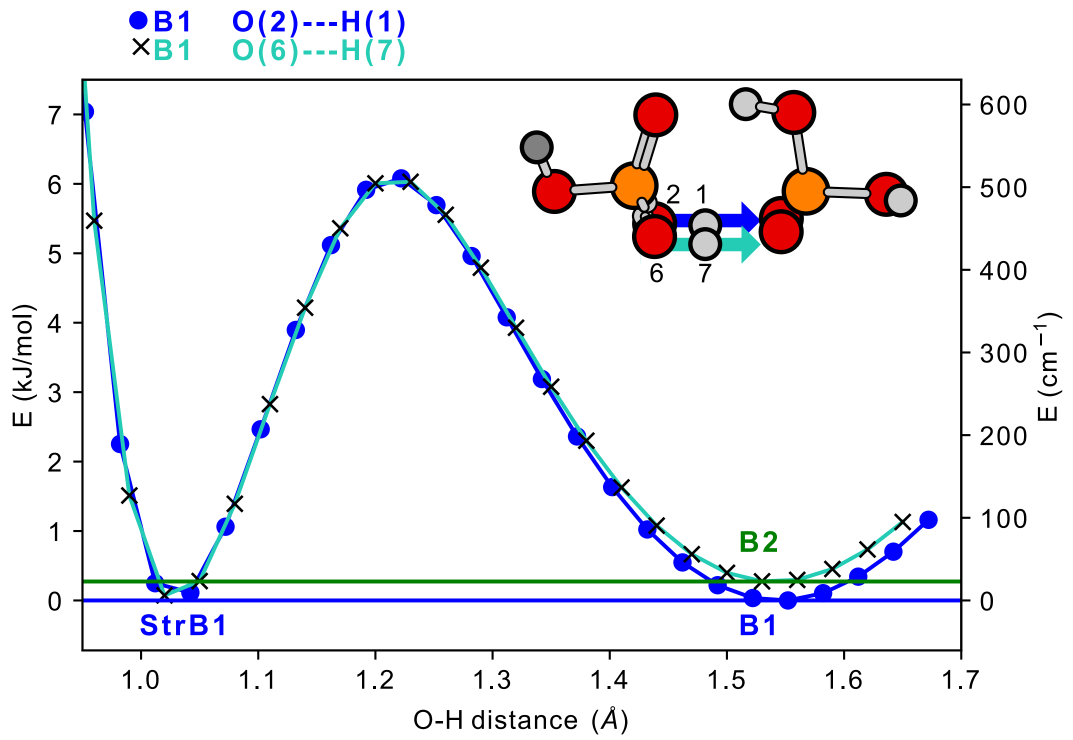

Figure S 5: Potential energy scans of the proton transfer coordinate that links B1 and B2 structures. The scans were computed at the B3LYP-D3(BJ)/aug- cc-pVTZ level of theory. It should be noted that a transfer can cause an isomerization between B1 and B2.

3. List of vibrational transitions

The experimentally observed vibrational transitions are summarized in Tables S2 and S3, alongside the corresponding calculated vibrational frequencies and modes obtained from calculations in both harmonic and anharmonic (VPT2) approximations. Harmonic normal modes were visualized to help correlate them to vibrational motions of specific atoms or groups of atoms. Additionally, in Fig. S6, the structure of interest A1 is displayed with the atoms labeled to aid the description of the modes in the Tables S2 and S3. Most of the modes are found to be delocalized and strongly coupled, which complicates a straightforward assignment. The mode descriptions provided in Tables S2 and S3 are intended to offer a general overview of the vibrational modes.

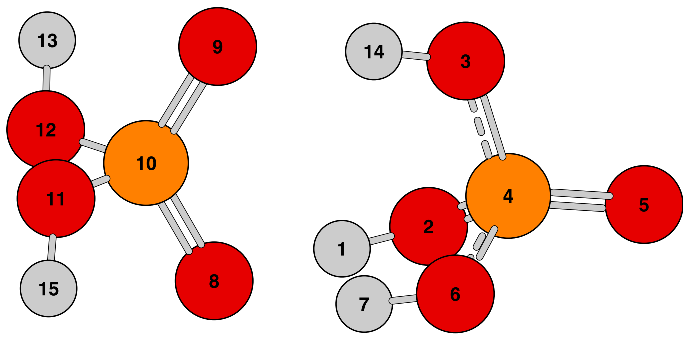

Optimized structure of A1 with atom labels. The labeling is intended to facilitate the interpretation of the vibrational mode descriptions presented in Tables S2 and S3.

Table S 2: List of the experimentally observed transitions of dPAD-H_5 and dPAD-D_5 in the mid-IR region in comparison of the calculated vibrational frequencies at the B3LYP-D3(BJ)/aug- cc-pV(T+d)Z in the harmonic approximation

$H_3PO_4H_2PO_4^-$

Experiment		B3LYP(BJ)			
label	freq. (cm^{-1})	#mode	frequency	intensity	assignment
a	830	21	816.2	54	sym. P-O11, P-O12 stretch + P-O-H1, P-O-H7 bend
		22	824.6	3	O3-H14-O9, H14 out of plane motion
		23	833.2	54	sym. P-O2, P-O3, P-O6 stretch
b	879	24	864.2	234	antisym. P-O11, P-O12 stretch
c	908	25	898.7	315	antisym. P-O2, P-O6 stretch
d	925	26	954.3	209	P-O3 stretch
e	965				
f	1025	27	1029.9	98	symmetric P-O-H13, P-O-H15 bend
		28	1044.9	90	antisym. P-O-H13, P-O-H15 bend
g	1095	29	1091.3	452	sym. O-P-O^- stretch
h	1210	30	1165.0	248	antisym. P-O-H1, P-O-H7 bend
		31	1231.2	243	sym. P-O-H1, P-O-H7 bend
i	1265	32	1254.7	116	P-O-H14 bend
		33	1273.8	182	O-P-O^- antisym stretching
j	1366	34	1379.7	278	P=O5 stretch + P-O-H14 bend

$D_3PO_4D_2PO_4^-$

Experiment		B3LYP(BJ)			
label	freq. (cm^{-1})	#mode	frequency	intensity	assignment
1	844	24	821.6	60	P-O-D15 bend + misc. P-O stretch
		25	824.9	14	P-O6, P-O2 stretch + P-O-D15, P-O-D13 bend
b'	875	26	830.0	143	misc. P-O stretch + P-O-D13 bend
		27	859.6	249	antisym. P-O11, P-O12 stretch
3	915	28	902.0	8	antisym. P-O-D1, D7, D14 bend
4	943	29	939.7	390	antisym. P-O2, P-O6 stretch
5	955	30	955.0	227	sym. P-O2, P-O6 stretch + sym. P-O-D1, P-O-D7 bend
e'	967	31	971.3	39	sym. P-O-D1, D7, D14 bend
g'	1102	32	1094.4	415	sym. O-P-O^- stretch
8	1228	33	1246.5	322	antisym. O-P-O^- stretch
9	1250				
10	1331	34	1320.7	345	P=O5 stretching

Table S 3: List of the experimentally observed transitions of dPAD-H_5 and dPAD-D_5 in the OH stretching (ν(OH)) region in comparison of the calculated vibrational frequencies at the B3LYP-D3(BJ)/aug- cc-pV(T+d)Z in the harmonic and anharmonic approximations. Vibrations are labeled as ν_s(OH) and ν_{as}(OH) to describe respectively, symmetric and anti-symmetric OH stretchings

$H_3PO_4H_2PO_4^-$

HeDrop	IRPD	B3LYP(BJ)				VPT2			
freq	freq	#	freq	intensity	assignment	#	freq	intensity	assignment
2855	2850	35	3163.1	1073	ν(OH14)	35	2828.5	919	Fund(35)
2908	2950					287	2970.7	77	Comb(6,35)[a]
2935	2920								
3160	3200	36	3403.8	61	ν_{as}(OH1, OH7)	36	3144.5	135	Fund(36)
		37	3479.2	1486	ν_s(OH1, OH7)	255	3165.5	637	Comb(5,36)[b]
						437	3181.3	43	Comb(11,35)[c]
						37	3324.8	517	Fund(37)
	3690	38	3839.9	126	ν_{as}(OH13, OH15)	38	3649.9	65	Fund(38)
		39	3841.3	7	ν_s(OH13, OH15)	39	3665.0	45	Fund(39)

$D_3PO_4D_2PO_4^-$

HeDrop	IRPD	B3LYP(BJ)				VPT2			
freq	freq	#	freq	intensity	assignment	#	freq	intensity	assignment
2190	2195	35	2310.6	571	ν(OD14)	35	2141.2	508	Fund(35)
2395	2398	36	2480.1	31	ν_{as}(OD1, OD7)	36	2343.8	40	Fund(36)
2495	2490	37	2533.4	809	ν_s(OD1, OD7)	37	2361.1	474	Fund(37)
2725	2720					255	2472.8	274	Comb(5,36)[d]
2725	2720	38	2793.6	80	ν_{as}(OD13, OD15)	38	2692.7	47	Fund(38)
		39	2795.5	5	ν_s(OD13, OD15)	39	2702.9	29	Fund(39)

[a] Mode 6 of frequency 157 cm^{-1}, intensity of 0.3 a.u., consists mainly of a rocking of the POO^- group dihydrogen phosphate anion

[b] Mode 5 of frequency 121 cm^{-1}, intensity of 32 a.u., consists mainly of a rocking of the dihydrogen phosphate anion

[c] Mode 11 of frequency 369 cm^{-1}, intensity of 1 a.u. consists mainly of a HO-P-OH siccoring motion of the phosphoric acid.

[d] Mode 5 of frequency 114 cm^{-1}, intensity of 5 a.u., consists mainly of a rocking of the dihydrogen phosphate anion

4. Mid IR spectra of the deuterated deprotonated dimer of phosphoric acid compared to calculations of the individual components

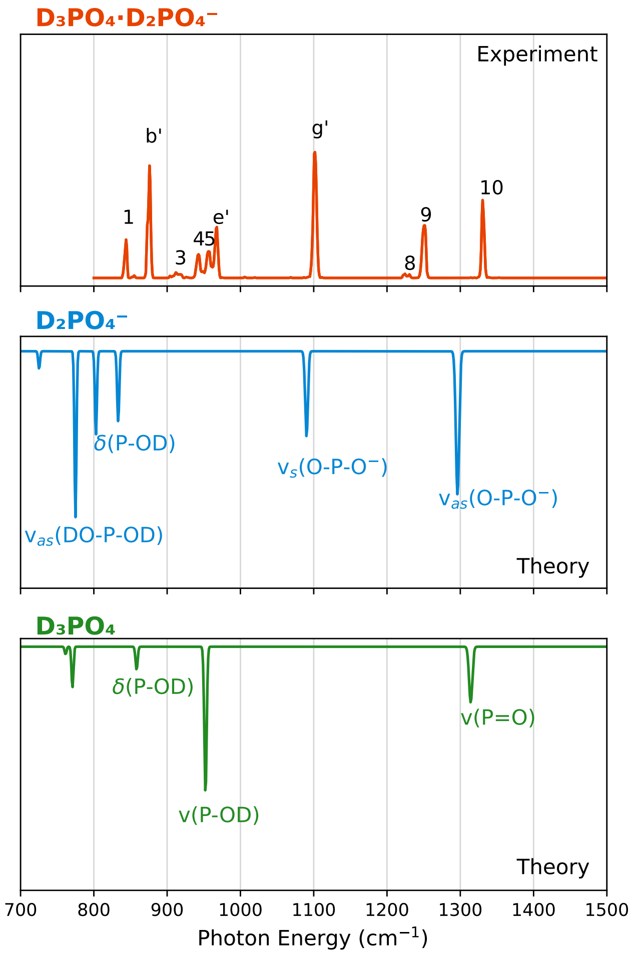

Figure S 6: IR action spectrum recorded using helium nanodroplets of the fully deuterated deprotonated dimer of phosphoric acid dPAD-H_5 compared to the calculated IR spectra of the two moieties, the deuterated neutral phosphoric acid (D_3PO_4) and the deuterated dihydrogen phosphate ($D_2PO_4^-$) anion, at the B3LYP-D3(BJ)/aug-cc-pV(T+d)Z level of theory in the harmonic approximation. No scaling factor is used.

5. Mid IR spectra of the deprotonated dimer of phosphoric acid in comparison of calculations at different levels of theory

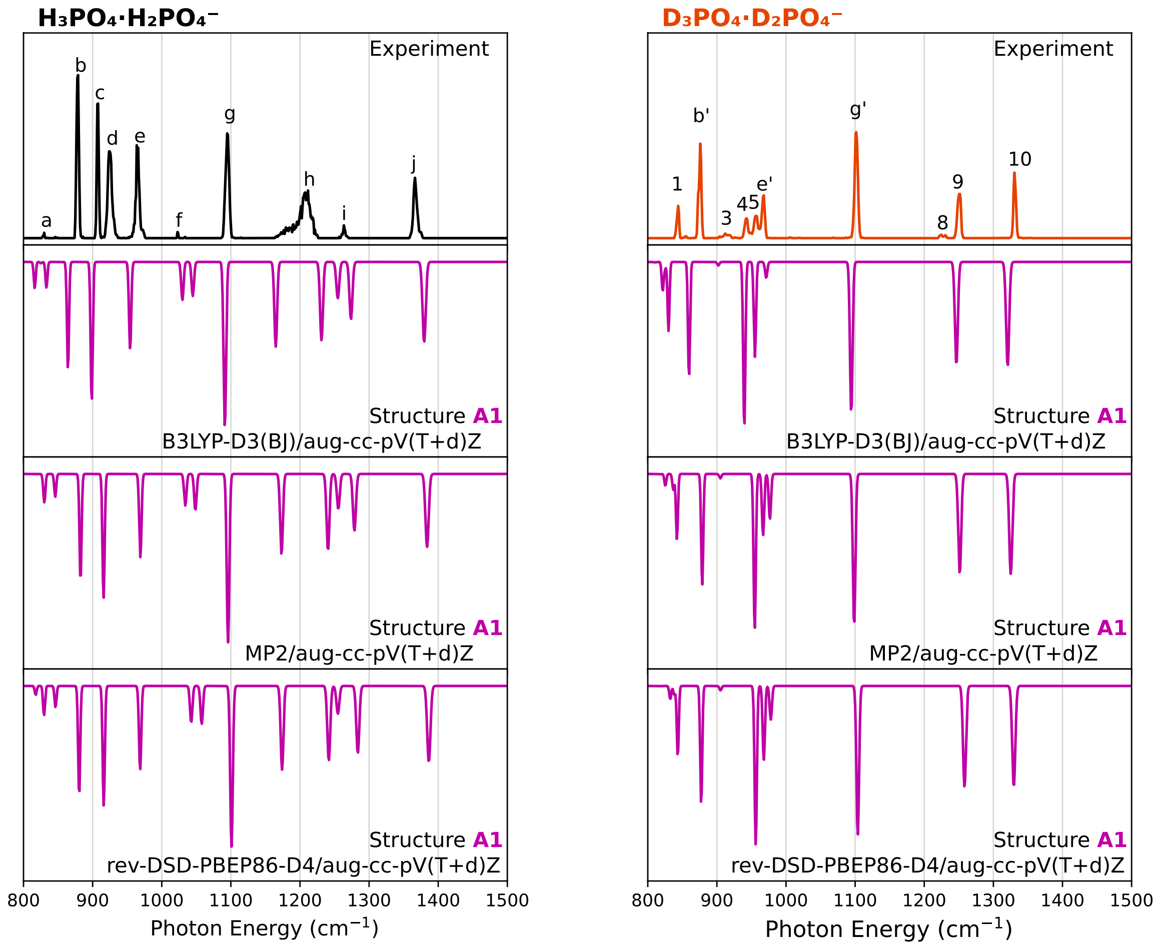

Figure S 7: IR action spectrum recorded using helium nanodroplets of the deprotonated dimer of phosphoric acid dPAD-H_5 (left panel) and its fully deuterated counterpart dPAD-D_5 (right panel) compared to the calculated IR spectra of structures A1 (magenta)at the B3LYP(BJ), MP2 and revDSD-PBEP86-D4 level of theory in the harmonic approximation.

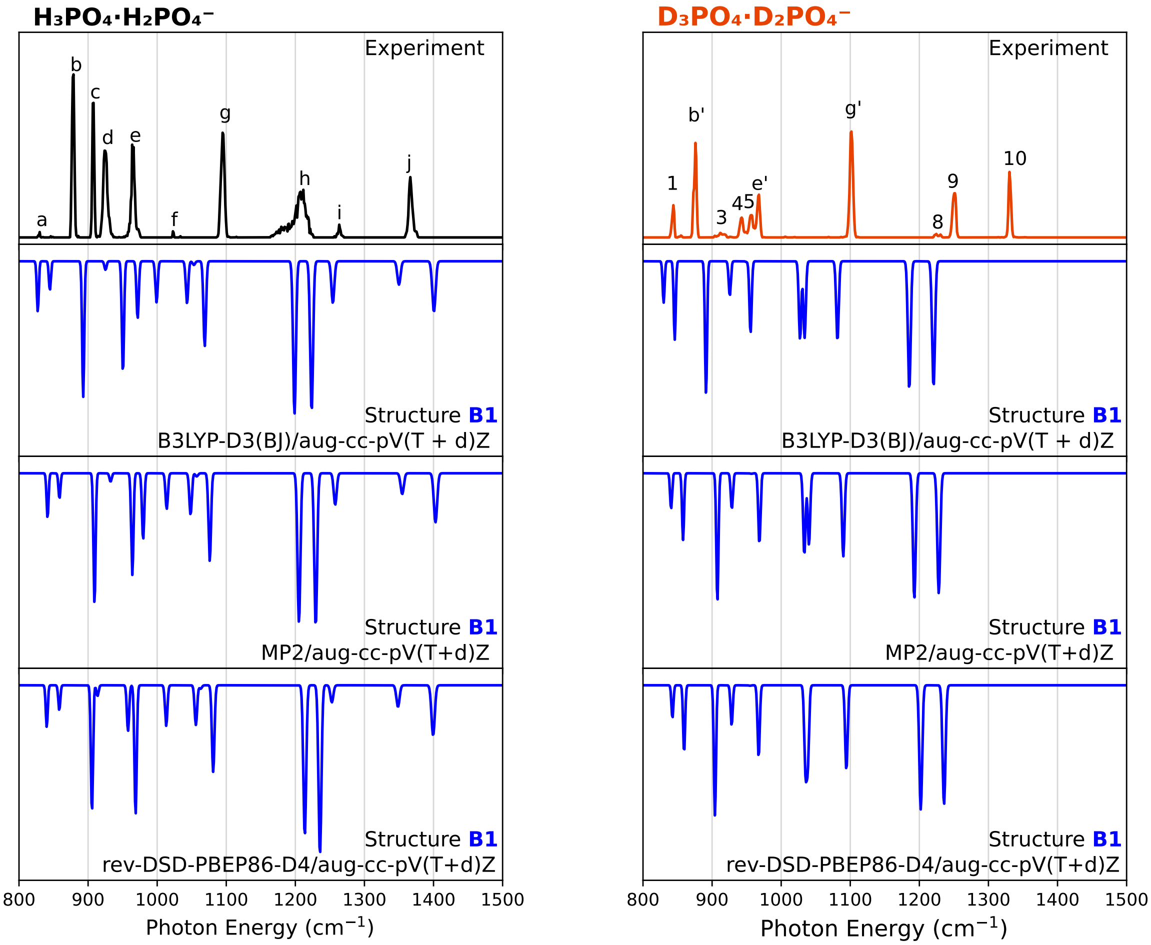

Figure S 8: IR action spectrum recorded using helium nanodroplets of the deprotonated dimer of phosphoric acid dPAD-H_5 (left panel) and its fully deuterated counterpart dPAD-D_5 (right panel) compared to the calculated IR spectra of structure B1 (blue) at the B3LYP(BJ), MP2 and revDSD-PBEP86-D4 level of theory in the harmonic approximation.

6. Spectra of the deprotonated dimer of phosphoric acid at the VPT2 level

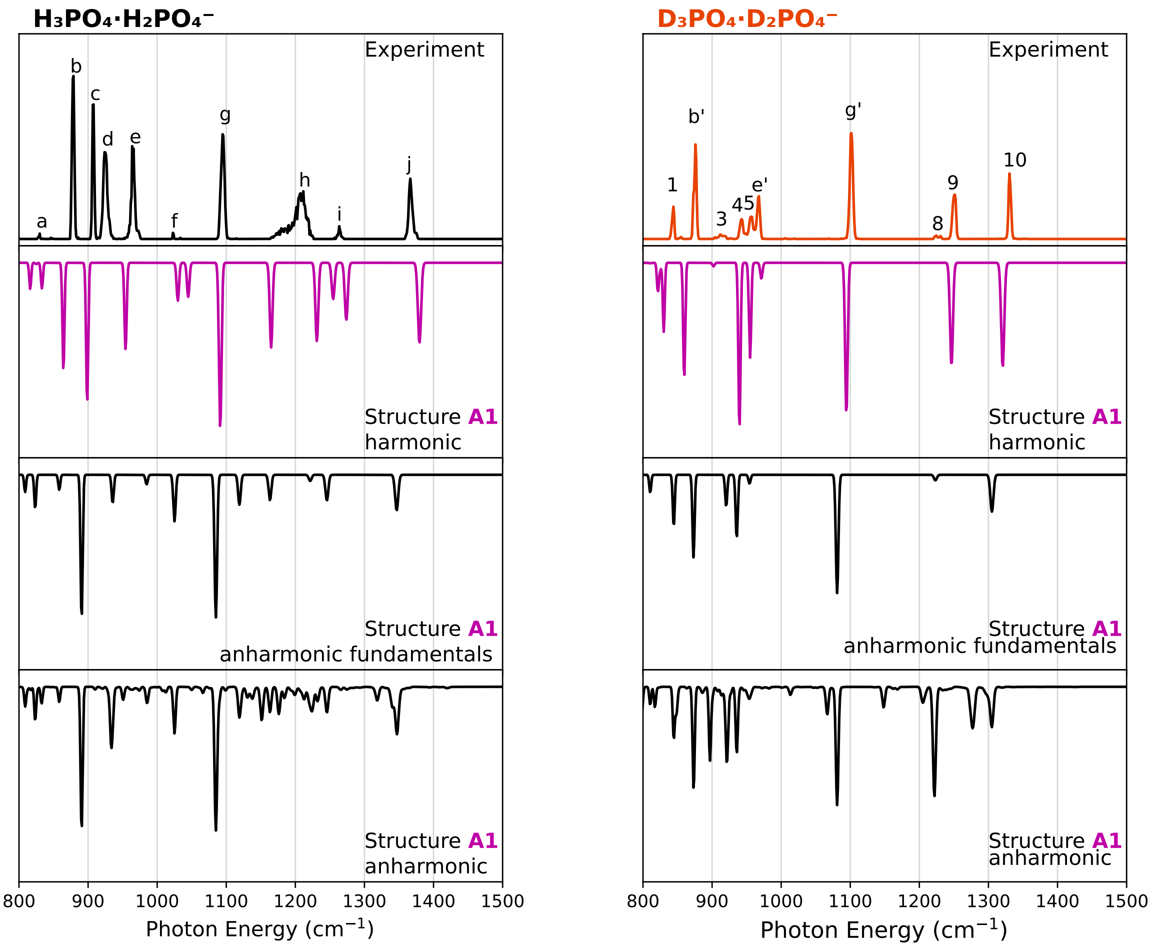

Figure S 9: IR action spectra recorded using helium nanodroplets of the deprotonated dimer of phosphoric acid dPAD-H_5 (left panel) and its fully deuterated counterpart dPAD-D_5 (right panel) compared to the calculated IR spectra of structure A1 at the B3LYP-D3(BJ)/aug- cc-pV(T+d)Z level of theory in the anharmonic approximation. The middle panel shows only the fundamental frequencies, and the bottom panel includes the overtones and combination bands.

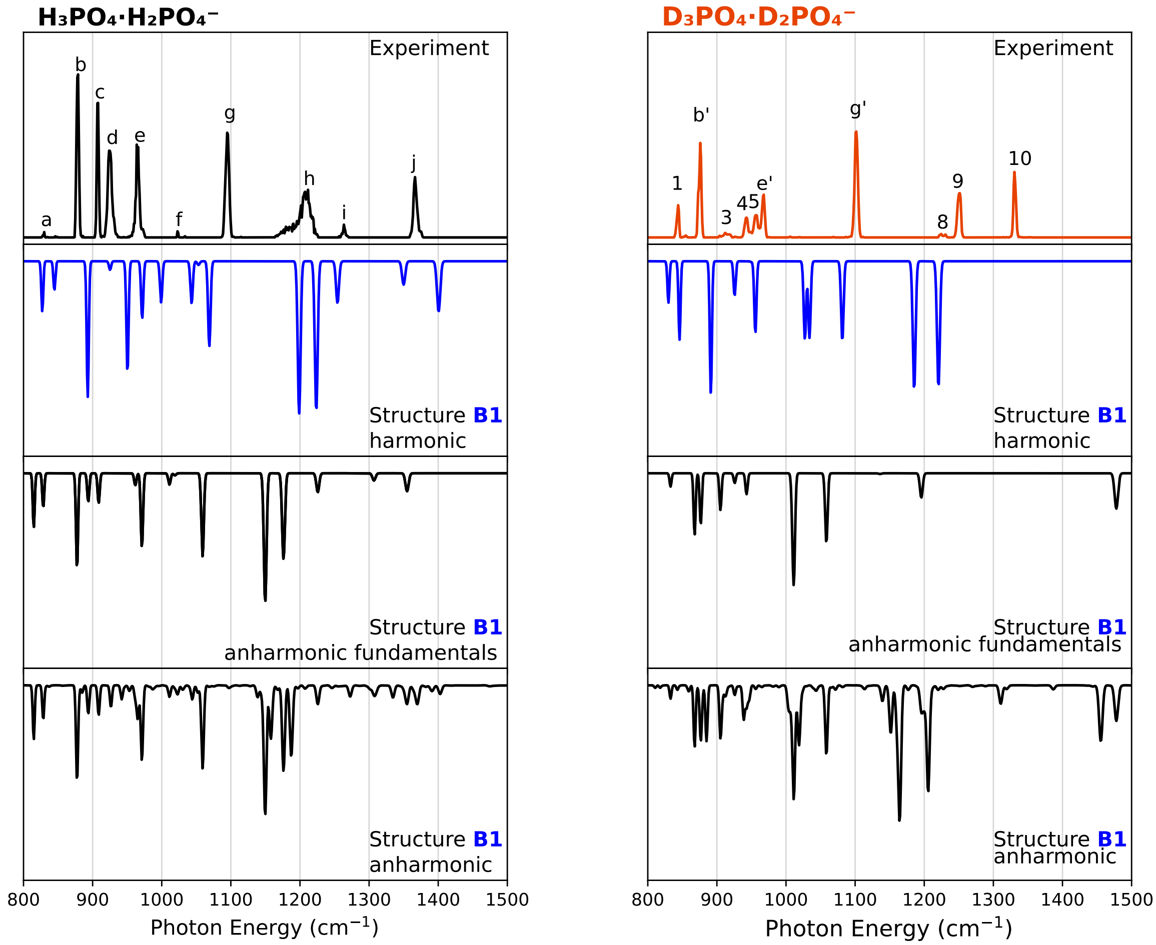

Figure S 10: IR action spectra recorded using helium nanodroplets of the deprotonated dimer of phosphoric acid dPAD-H_5 (left panel) and its fully deuterated counterpart dPAD-D_5 (right panel) compared to the calculated IR spectra of structure B1 at the B3LYP-D3(BJ)/aug- cc-pV(T+d)Z level of theory in the anharmonic approximation. The middle panel shows only the fundamental frequencies, and the bottom panel includes the overtones and combination bands.

7. Spectrum of $H_2PO_4^-$ and the effect of anharmonicity

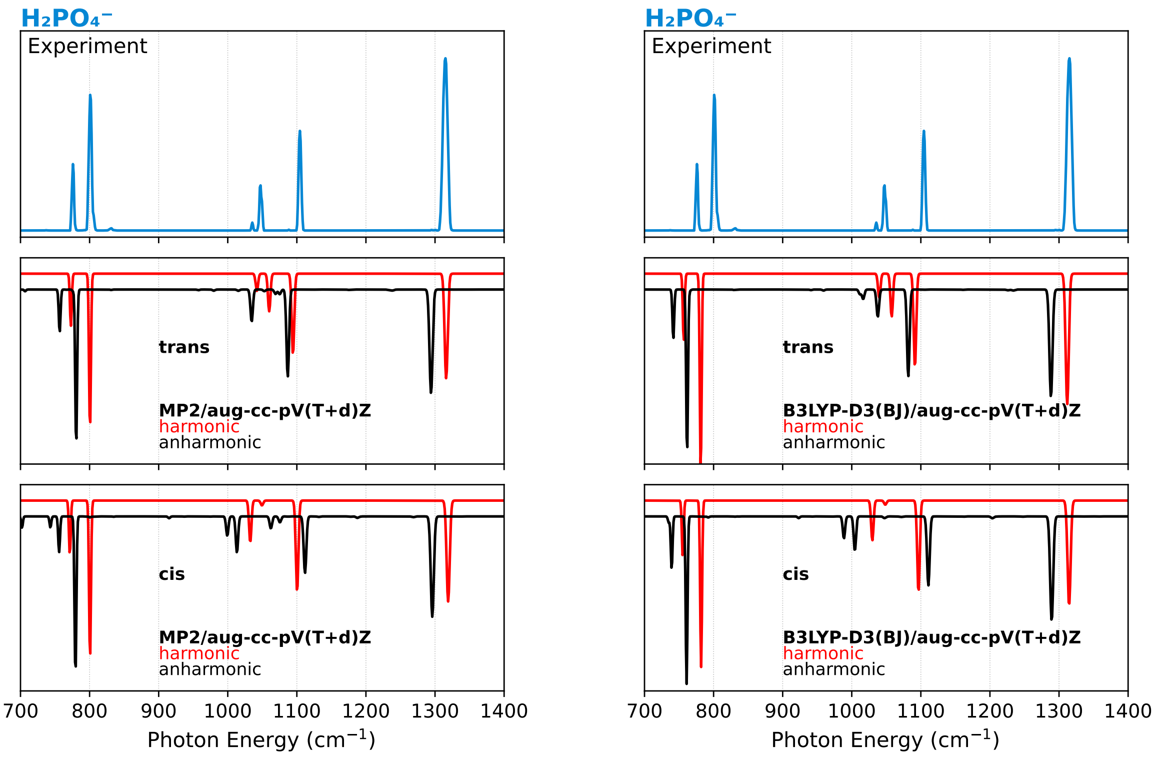

Figure S 11: IR action spectrum recorded using helium nanodroplets of the dihydrogen phosphate compared to the calculated IR spectra for both OH groups at cis and trans conformations using the B3LYP-D3(BJ)/aug- cc-pV(T+d)Z and MP2/aug-cc-pV(T+d)Z level of theory in the harmonic and anharmonic approximations.

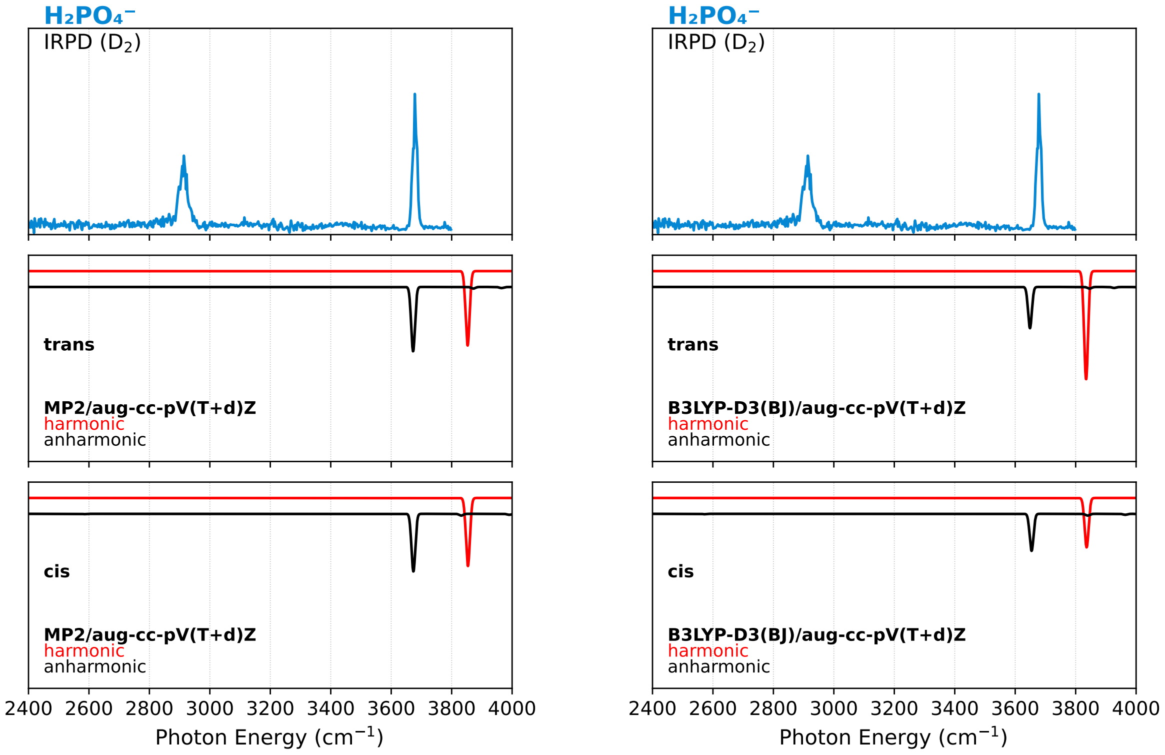

Figure S 12: IRPD spectrum recorded using D_2 tagging of the dihydrogen phosphate compared to the calculated IR spectra for both OH groups at cis and trans conformations using the B3LYP-D3(BJ)/aug- cc-pV(T+d)Z and MP2/aug-cc-pV(T+d)Z level of theory in the harmonic and anharmonic approximations. The weak band at 2900 cm^{-1} in the experimental spectrum is assigned to the D_2 stretching vibration.

8. Cartesian coordinates of the optimized structures

Cartesian coordinates and electronic energies obtained from optimization calculations using B3LYP-D3(BJ)/aug-cc-pV(T+d)Z level of theory for the different molecular and anionic structures discussed in this work.

$H_3PO_4{\cdot}H_2PO_4^-$, structure A1

Electronic energy (Hartrees): -1288.28412

```
-1 1
 H                  0.51931500    0.05026400    1.46512000
 O                  1.44040500   -0.28914900    1.43423800
 O                  1.44417000   -1.07308500   -0.99437700
 P                  2.07815800   -0.01085000   -0.00829100
 O                  3.54172800   -0.04747700   -0.01428300
 O                  1.47214900    1.41228600   -0.42052400
 H                  0.53899800    1.47451700   -0.11781900
 O                 -0.95036600    0.91514500    0.76556100
 O                 -1.22491300   -0.99562800   -0.97642800
 P                 -1.82808100   -0.01543100   -0.02753200
 O                 -2.90494700    0.90067000   -0.82614900
 O                 -2.76089700   -0.83408100    1.02296900
 H                 -2.80821000   -1.75102600    0.73621100
 H                  0.44822700   -1.07355400   -1.02723200
 H                 -2.90810300    1.78456800   -0.44700500
```

$H_3PO_4 \cdot H_2PO_4^-$, structure A2

Electronic energy (Hartrees): -1288.28363

```
-1 1
 H                  0.53456400   -0.13844400    1.46523500
 O                  1.45479500   -0.47520500    1.38278000
 O                  1.43108700   -0.93972600   -1.12569200
 P                  2.07935000   -0.01365300   -0.01678900
 O                  3.54264400   -0.05469800   -0.04602700
 O                  1.47594100    1.45163600   -0.23809300
 H                  0.54880700    1.48594800    0.08679600
 O                 -0.92872100    0.79371200    0.91534800
 O                 -1.23950800   -0.86824000   -1.05624200
 P                 -1.82551500    0.01286800    0.00628700
 O                 -2.84446900    1.07904100   -0.66750200
 O                 -2.82282000   -0.90123300    0.91366100
 H                 -2.72721000   -1.81583400    0.63193000
 H                  0.43537300   -0.93796000   -1.13986000
 H                 -3.15064300    0.73575400   -1.51242300
```

$H_3PO_4 \cdot H_2PO_4^-$, structure B1

Electronic energy (Hartrees): -1288.28394

```
-1 1
 H                 0.30933800   -0.25529800   -1.56151100
 O                 1.33255300   -0.28235100   -1.45557000
 O                 1.31137700    1.37679300    0.51262000
 P                 1.73918600    0.03704000    0.02262600
 O                 3.34214500   -0.08707100   -0.00487400
 O                 1.30839800   -1.16366500    0.93150800
 H                 0.28289400   -1.20786200    1.00806400
 O                -1.25291600   -1.06314200    0.95848700
 O                -1.22840100   -0.16686400   -1.45615700
 P                -1.73667800   -0.04504100   -0.04826400
 O                -1.47998100    1.43974100    0.51041000
 O                -3.35417000   -0.09037500   -0.08047900
 H                -3.66405100   -0.46715900    0.74843700
 H                 3.71192400    0.74964500    0.29186000
 H                -0.50978100    1.59616400    0.57016900
```

$H_3PO_4 \cdot H_2PO_4^-$, structure B2

Electronic energy (Hartrees): -1288.28386

-1 1

H	-0.31718400	-0.50702300	1.48786500
O	-1.34061900	-0.49597900	1.37517400
O	-1.29576300	1.48554000	-0.27147200
P	-1.74074000	0.09530900	-0.02594800
O	-3.34312700	0.00147900	-0.02356400
O	-1.31634100	-0.94295100	-1.12875900
H	-0.29122300	-0.98588000	-1.21394700
O	1.24437100	-0.88181000	-1.13407800
O	1.21917400	-0.43957400	1.40303000
P	1.73483600	-0.06864100	0.04185300
O	1.49806100	1.49502300	-0.23885300
O	3.35129700	-0.13940900	0.07186200
H	3.66425400	-0.34191700	-0.81480900
H	-3.63447800	-0.89874600	0.15307700
H	0.53078500	1.67498100	-0.27746000

H_3PO_4 (C_3)

Electronic energy (Hartrees): -644.37

```
0 1
 H                  0.49095400    2.08454500   -0.08002300
 O                  0.00000000    1.42814200   -0.58687800
 O                 -1.23680700   -0.71407100   -0.58687800
 P                  0.00000000    0.00000000    0.11359800
 O                  0.00000000    0.00000000    1.57764700
 O                  1.23680700   -0.71407100   -0.58687800
 H                  1.55979200   -1.46745100   -0.08002300
 H                 -2.05074600   -0.61709400   -0.08002300
```

$H_2PO_4^-$ (C_2)

Electronic energy (Hartrees): -643.84

```
-1 1
 H                  1.54941100   -0.93008700   -0.96726700
 O                  1.28118500   -0.01028000   -0.88547200
 O                  0.00000000    1.32326900    0.85032100
 P                  0.00000000    0.00000000    0.16646400
 O                  0.00000000   -1.32326900    0.85032100
 O                 -1.28118500    0.01028000   -0.88547200
 H                 -1.54941100    0.93008700   -0.96726700
```
